# Building better brains: the pleiotropic function of neurotrophic factors in postnatal cerebellar development

**DOI:** 10.3389/fnmol.2023.1181397

**Published:** 2023-05-12

**Authors:** Pia Boxy, Anders Nykjær, Lilian Kisiswa

**Affiliations:** ^1^Department of Biomedicine, Aarhus University, Aarhus, Denmark; ^2^Danish Research Institute of Translational Neuroscience (DANDRITE)–Nordic EMBL Partnership for Molecular Medicine, Aarhus University, Aarhus, Denmark; ^3^The Danish National Research Foundation Center, PROMEMO, Aarhus University, Aarhus, Denmark

**Keywords:** cerebellum, neurotrophic factors, signaling/signaling pathways, development, developmental disorder

## Abstract

The cerebellum is a multifunctional brain region that controls diverse motor and non-motor behaviors. As a result, impairments in the cerebellar architecture and circuitry lead to a vast array of neuropsychiatric and neurodevelopmental disorders. Neurotrophins and neurotrophic growth factors play essential roles in the development as well as maintenance of the central and peripheral nervous system which is crucial for normal brain function. Their timely expression throughout embryonic and postnatal stages is important for promoting growth and survival of both neurons and glial cells. During postnatal development, the cerebellum undergoes changes in its cellular organization, which is regulated by a variety of molecular factors, including neurotrophic factors. Studies have shown that these factors and their receptors promote proper formation of the cerebellar cytoarchitecture as well as maintenance of the cerebellar circuits. In this review, we will summarize what is known on the neurotrophic factors’ role in cerebellar postnatal development and how their dysregulation assists in developing various neurological disorders. Understanding the expression patterns and signaling mechanisms of these factors and their receptors is crucial for elucidating their function within the cerebellum and for developing therapeutic strategies for cerebellar-related disorders.

## Introduction

The cerebellum, or ¨little brain¨, is well-known for its sensorimotor function and its role in movement coordination (Gao et al., 1996; Strick et al., 2009). Nevertheless, growing evidence indicates that the little brain is also involved in higher-order cognitive processing, including spatial learning, attention, language, reward, emotion, social behavior and memory (Ito, 2006; Schmahmann and Caplan, 2006; Ito, 2008; Stoodley, 2012; Koziol et al., 2014; Adamaszek et al., 2017; Wagner et al., 2017; Carta et al., 2019; Kostadinov et al., 2019). Several neuroimaging and lesion studies have shown cerebellar aberrations to account for changes in affective and cognitive behavior, collectively termed the “cerebellar cognitive affective syndrome” with deficits in executive function, emotion regulation, and working memory (Schmahmann and Sherman, 1998). Aberrant cerebellar functionality and cerebellar-only genetic alterations have been implicated in numerous neuropsychiatric disorders, such as schizophrenia and bipolar disorder, and neurodevelopmental disorders, including attention deficit hyperactivity disorder (ADHD) and autism spectrum disorder (ASD) (Allen et al., 2004; Peter et al., 2016; Stoodley, 2016; Sathyanesan et al., 2019). This is mainly because cerebellar learning, complementing its role in motor control, also has a bottom-up influence on cognitive functions through extensive interconnections between the deep cerebellar nuclei and limbic brain structures (Snider et al., 1976; Carta et al., 2019; Frontera et al., 2020). Hence, if genetically predisposed, environmental perturbations throughout most of the cerebellar development may impair neuronal maturation and synapse formation, and lead to incorrect circuit wiring.

The cerebellum is a highly conserved brain structure that has an extensive and elaborate development, which starts in humans at gestational week 7 (E13 in rodents) and ends around 12 months postnatal (P21 in rodents) (Larramendi and Victor, 1967; Altman, 1972; Sathyanesan et al., 2019). For this review we will focus on the postnatal development of the cerebellum since the embryonic development has been covered in plenum by other reviews (Leto et al., 2016; Sathyanesan et al., 2019; Amore et al., 2021). During early postnatal stages, the dendritic complexity of Purkinje cells (PCs) develops extensively in terms of both branching and arbor length, resulting in the thickening of the molecular layer (Leto et al., 2016). Granule cell progenitors (GCPs), on the one hand, form the temporary external granule layer (EGL) in which they actively proliferate and expand. Consequently, the immature GCPs exit the cell cycle, commence differentiation, after which they migrate radially until they reach their destination within the internal granule layer (IGL) and become mature cerebellar granule neurons (CGNs). During this process, there is both extension of the CGN axons, namely the parallel fibers, and growth of the CGN dendrites (Consalez et al., 2021). The inhibitory interneurons (IN), on the other hand, originate from a pool of progenitors located in the cerebellar white matter (WM) that give rise to a variety of glial cells, including astrocytes, oligodendrocytes, and Bergmann glia (BG), as well as several types of interneurons. These progenitors migrate through the WM into the cerebellar cortex and subsequently differentiate into mature glia and INs (Zhang and Goldman, 1996). Lastly, synaptogenesis and target innervation of the PC axons occurs followed by synaptic and dendritic pruning (Leto et al., 2016).

**Figure 1:**
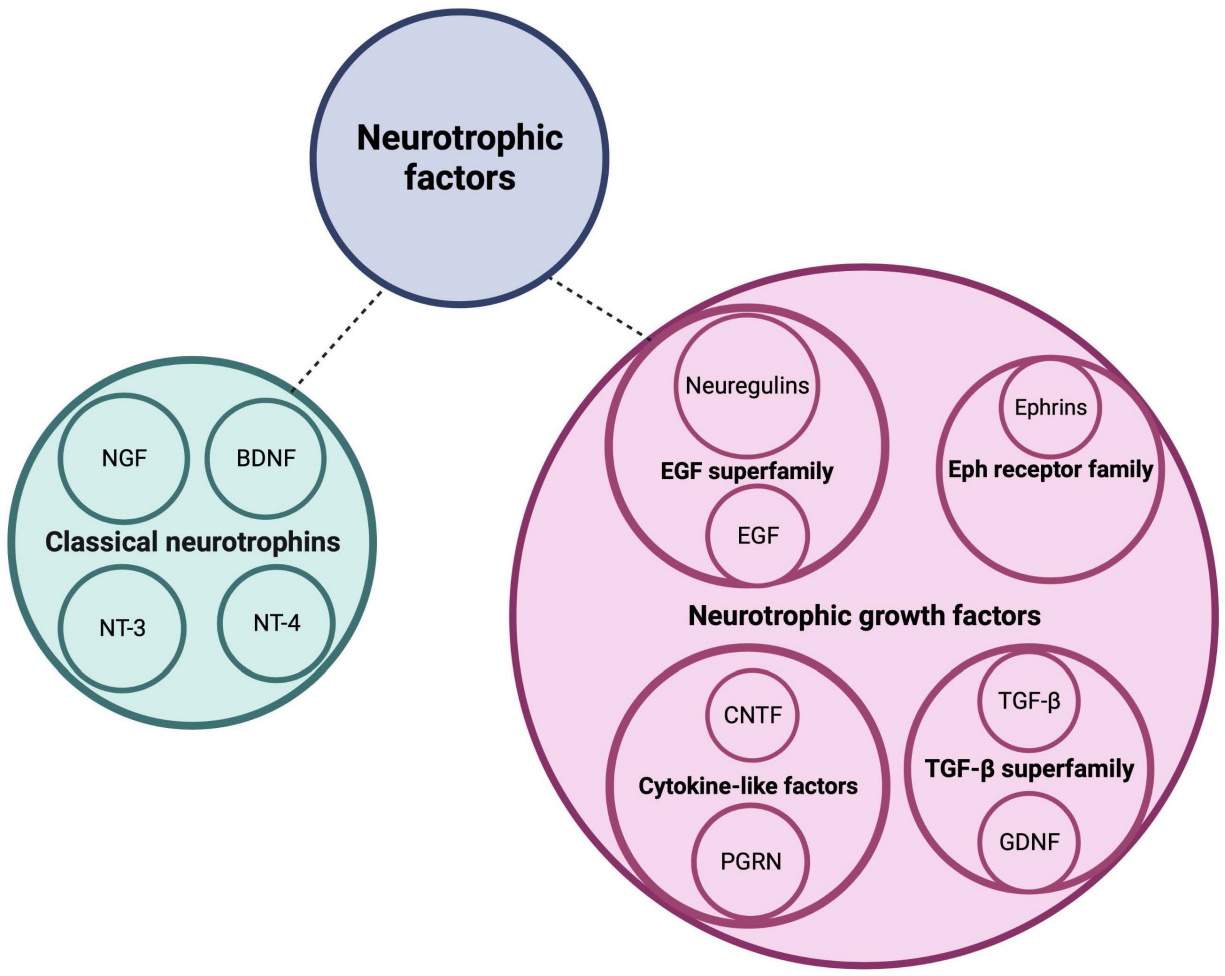
Neurotrophic factors regulate postnatal cerebellar development. This schematic diagram depicts the families of neurotrophic factors that regulate survival, differentiation, and migration of cerebellar neurons as well as neurite circuit formation and maintenance. BDNF, brain-derived neurotrophic factor; CNTF, ciliary neurotrophic factor; EGF, epidermal growth factor; GDNF, glial-derived neurotrophic factor; NGF, nerve growth factor; NT-3, neurotrophin-3; NT-4, neurotrophin-4; PGRN, progranulin; TGF-b, transforming growth factor beta. Figure produced in BioRender.

Cerebellar development is a complex process that is regulated by a variety of signaling molecules and growth factors, both in a cell-autonomous and non-cell-autonomous manner. One category of growth factors that play a crucial role in cerebellar development and circuit formation is the neurotrophic factors (Figure 1). This includes the classical neurotrophins brain-derived neurotrophic factor (BNDF), nerve growth factor (NGF), neurotrophin-3 (NT-3) and neurotrophin-4 (NT-4), that are widely and timely expressed in different regions of the central and peripheral nervous system. Neurotrophins are key players during nervous system development where they regulate neurogenesis, morphogenesis, synaptogenesis, and cell maintenance, as well as during adulthood, in processes of cellular survival or death and, synaptic plasticity (Huang and Reichardt, 2001). The neurotrophins occur as both their secreted precursor state and their cleaved mature form (Lee et al., 2001). Mature neurotrophins bind preferentially to the high-affinity Trk receptors, a family of transmembrane tyrosine kinases, to regulate neuronal survival and differentiation (Lu et al., 2005). Activation of Trks initiates several signaling cascades, including the mitogen-activated protein kinase (MAPK) cascade, the phosphatidylinositol-3-kinase (PI3K) cascade, and the protein kinase C (PKC)-phospholipase-Cγ (PLC-γ) cascade (Klesse and Parada, 1999; Reichardt, 2006). Proneurotrophins, however, preferentially interact with the low-affinity and nonselective p75 neurotrophin receptor (p75^NTR^) of the tumor necrosis factor (TNF) receptor superfamily in a complex with members of the sortilin receptor family to regulate cell death via the c-Jun N-terminal kinase (JNK) or caspase-3 pathway (Yoon et al., 1998; Friedman, 2000; Hempstead and Salzer, 2002; Nykjaer et al., 2004). NGF binds TrkA, BDNF and NT-4 bind TrkB, and NT-3 binds primarily TrkC (Chao and Hempstead, 1995). In addition to the prototypical neurotrophins, there are numerous growth factors that also play a major role in proper brain development (Figure 1). These include ciliary neurotrophic factor (CNTF), ephrins, epidermal growth factor (EGF), glial cell line-derived neurotrophic factor (GDNF), neuregulins, progranulin (PGRN) and transforming growth factor (TGF-β). By binding to their respective receptors, they provide survival, differentiation, migration, maturation, and circuit formation signals to the developing nervous system (Abe et al., 1991; Lärkfors et al., 1994; Mount et al., 1995; Ozaki et al., 2000; Rodger et al., 2012; Araujo et al., 2016; Uesaka et al., 2018). CNTF binds the CNTF receptor (CNTFR), ephrins bind to the Eph tyrosine kinase receptors, EGF binds to the EGF receptor (EGFR) or receptor homologs, GDNF binds predominantly GFRα1 in complex with the RET receptor, neuregulins bind the ErbB family of receptors, PGRN binds TNF receptors as well as the sortilin receptor, and lastly, TGF-β isoforms binds to the TGF-β type I, II, and III receptors (TβRI, TβRII, and TβRIII) (Cheifetz et al., 1988; Treanor et al., 1996; Chang et al., 1997; Doré et al., 1998; Wieduwilt and Moasser, 2008; Hu et al., 2010; Tang et al., 2011; Fantone et al., 2020). Binding of these growth factors to their respective receptors activates various signaling pathways including the p38 and JNK-MAPK, Jak–STAT, Ras-MAPK, and PI3K-Akt signaling pathways (Bonni et al., 1993; Oh et al., 1998; Wong and Guillaud, 2004; Murphy and Bielby-Clarke, 2008; Paratcha and Ledda, 2008; Wee and Wang, 2017; Fantone et al., 2020; Wang et al., 2022).

**Figure 2:**
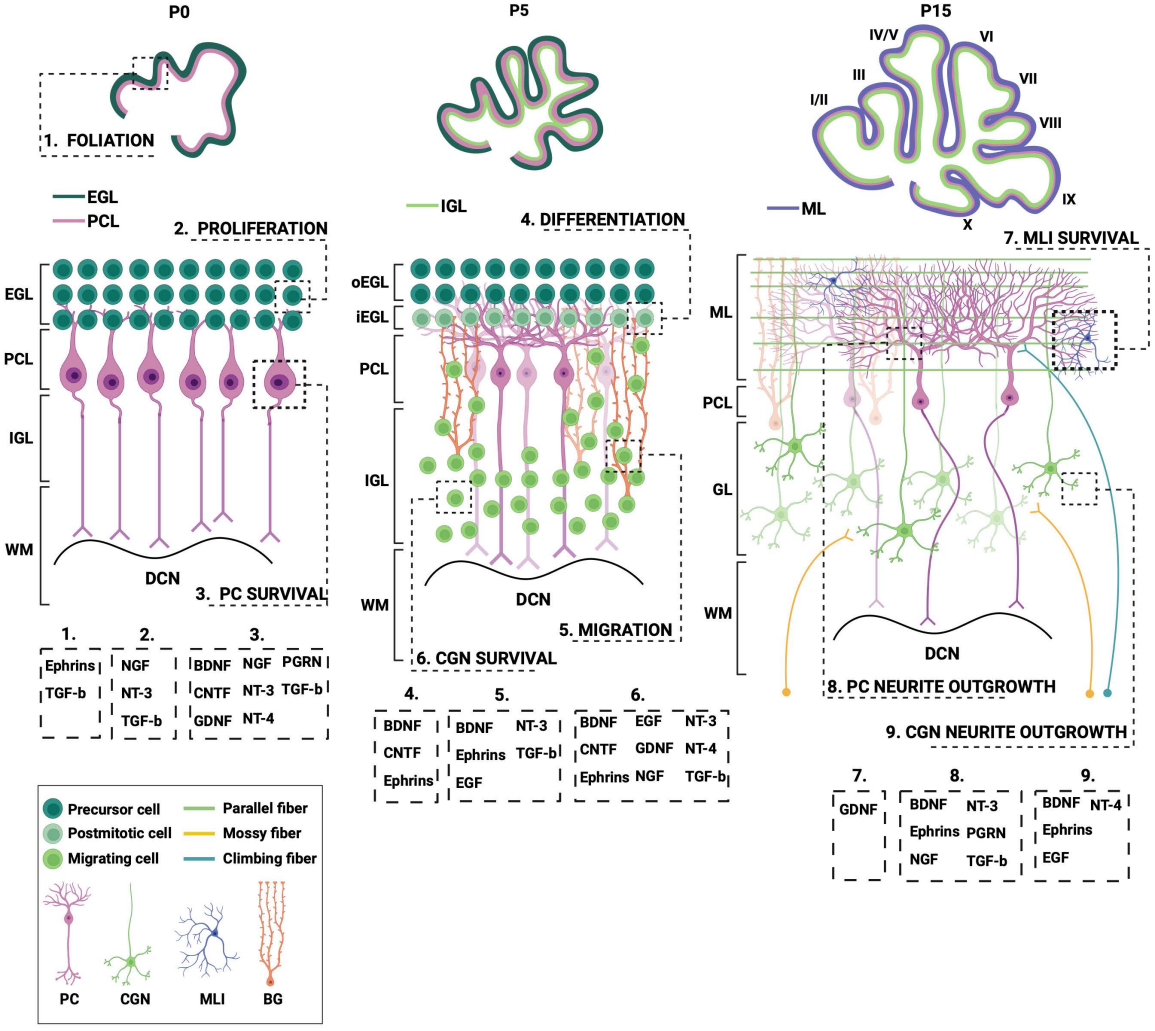
Different processes during postnatal cerebellar development require neurotrophic action. This schematic illustration depicts several stages of postnatal cerebellar development that involve the neurotrophic factors. EGL, external granule layer; oEGL, outer EGL; iEGL, inner EGL; PCL; Purkinje cell layer; ML, molecular layer; (I)GL, (internal) granule layer; WM, white matter; DCN, deep cerebellar nuclei; PC, Purkinje cell; CGN; cerebellar granule cell; MLI, molecular layer interneuron; BG, Bergmann glia; BDNF, brain-derived neurotrophic factor; CNTF, ciliary neurotrophic factor; EGF, epidermal growth factor; GDNF, glial-derived neurotrophic factor; NGF, nerve growth factor; NT-3, neurotrophin-3; NT-4, neurotrophin-4; PGRN, progranulin; TGF-b, transforming growth factor beta. Figure produced in BioRender.

In this review, we will summarize what is currently known about the spatiotemporal expression of the different neurotrophic factors and their receptors within the cerebellar system during postnatal development (Figure 2; Table 1). Additionally, we will outline the neurotrophic factors’ diverse cellular functionalities and some of the downstream signaling mechanisms that require neurotrophic expression and activity. Lastly, we will highlight how aberrations in both neurotrophic expression and function affect the cerebellar cytoarchitecture and what it implicates for several neurodevelopmental disorders (Table 2).

**Table 1:** Function of neurotrophic factors during postnatal cerebellar development.

Neurotrophic factors	Cerebellar function	References
BDNF	CGN survival	Bulleit and Hsieh (2000), Kubo et al. (1995), Leeds et al. (2005), Lindholm et al. (1993), Nonomura et al. (1996), Ortega et al. (2010), Sanchez-Perez et al. (2005), Shimoke et al. (1997), Skaper et al. (1998), Tong and Perez-Polo (1998), Zirrgiebel et al. (1995), and Koshimizu et al. (2010)
	CGN migration	Borghesani et al. (2002), Kokubo et al. (2009), and Zhou et al. (2007)
	CGN neurite outgrowth	Gao et al. (1995), Nonomura et al. (1996), and Tanaka et al. (2000)
	PC survival	Lärkfors et al. (1996), Rakotomamonjy and Goumari (2019), and Morrison and Mason (1998)
	PC neurite outgrowth	Schwartz et al. (1997), Shimada et al. (1998), and Yamashita et al. (2011)
	Circuit wiring	Bosman et al. (2006), Carter et al. (2002), Minichiello (1996), Rico et al. (2002), Schwartz et al. (1997), Shinoda et al. (2019), Chen et al. (2016), Bao et al. (1999), Seil (1999), Seil and Drake-Baumann (2000), Rabacchi et al. (1999), Choo et al. (2017), Ichise et al. (2000), Kano et al. (1997), and Abe et al. (2012)
CNTF	CGN survival	de Luca et al. (1996a)
	PC survival	Lärkfors et al. (1994)
	Astrocyte differentiation	Okano-Uchida et al. (2013)
Ephrins	CGN survival	Karam et al. (2000) and Sentürk et al. (2011)
	CGN migration	Yacubova and Komuro (2002)
	CGN neurite outgrowth	Karam et al. (2000), Sentürk et al. (2011), Moreno-Flores et al. (2002), and Wang et al. (2007)
	PC neurite outgrowth	Karam et al. (2000), Heintz et al. (2016), and Saywell et al. (2014)
	Circuit wiring	Lackey and Sillitoe (2020)
	Cerebellar foliation	Karam et al. (2000) and Rogers et al. (1999)
EGF	CGN survival	Abe et al. (1991, 1992), Morrison et al. (1988), Yamada et al. (1997), Gunn-Moore and Tavaré (1998) and Leutz and Schachner (1981)
	CGN migration	Carrasco et al. (2003), and Martinez et al. (2011)
	CGN neurite development	Abe et al. (1991, 1992), Morrison et al. (1988), and Yamada et al. (1997)
	NSC proliferation	Okano-Uchida et al. (2013) and Leutz and Schachner (1981)
GDNF	CGN survival	Subramaniam et al. (2008)
	PC survival	Mount et al. (1995)
	PC neurite outgrowth	Mount et al. (1995)
	MLI survival	Sergaki and Ibáñez (2017)
NGF	CGN survival	Legrand and Clos (1991), Muller et al. (1994), Khursigara et al. (2001), Kisiswa et al. (2018), and Vicario et al. (2015)
	PC survival	Cohen-Cory et al. (1991), Florez-McClure et al. (2004), Legrand and Clos (1991), and Mount et al. (1998)
	PC neurite outgrowth	Cohen-Cory et al. (1991), Legrand and Clos (1991), and Mount et al. (1998)
	Circuit wiring	Numakawa et al. (2003)
Neuregulins	Circuit wiring	Rieff and Corfas (2006), Ozaki (2001), Ozaki et al. (2000), Xie et al. (2004), Xie et al. (2007), Rieff et al. (1999), Ozaki et al. (2004), Gajendran et al. (2009), and Fenster et al. (2012)
NT-3	CGN survival	Katoh-Semba et al. (2000), Bates et al. (1999), Joo et al. (2014), Bates et al. (1999), Joo et al. (2014), Katoh-Semba et al. (2000), Kubo et al. (1995), and Shimoke et al. (1997)
	CGN differentiation	Doughty et al. (1998), Neveu and Arenas (1996), Takumi et al. (2005), Minichiello (1996), Zanin et al. (2016), Zanin and Friedman (2022), Zanin et al. (2019), and Segal et al. (1992)
	CGN migration	Neveu and Arenas (1996)
	PC survival	Lärkfors et al. (1996) and Mount et al. (1998)
	PC neurite outgrowth	Joo et al. (2014), Neveu and Arenas (1996), and Tepper et al. (2020)
	Circuit wiring	Sadakata et al. (2014), Shinoda et al. (2019), and Sherrard and Bower (2002)
NT-4	CGN survival	Proenca et al. (2016), Gao et al. (1995), Kubo et al. (1995), Shimoke et al. (1997), and Skaper et al. (1998)
	CGN neurite outgrowth	Gao et al. (1995)
	PC survival	Proenca et al. (2016), Lärkfors et al. (1996), and Morrison et al. (1988)
	Circuit wiring	Sadakata et al. (2014), Shinoda et al. (2019), and Sherrard and Bower (2002)
PGRN	PC survival	Wang et al. (2022)
	PC neurite outgrowth	Matsuwaki et al. (2015)
	Circuit wiring	Uesaka et al. (2018)
TGF-b	CGN survival	Wang et al. (2011), Elvers et al. (2005), de Luca et al. (1996b), Brown (1999), Constam et al. (1994), and Kane et al. (1996)
	CGN migration	Wang et al. (2011)
	PC survival	Zhou et al. (2003) and Wang et al. (2011)
	PC neurite outgrowth	Wang et al. (2011)
	Circuit wiring	Araujo et al. (2016), Ondáčová et al. (2017)
	Cerebellar foliation	Wang et al. (2011)

CGN, cerebellar granule neuron; PC, Purkinje cell; MLI, molecular layer interneuron; NSC, neuronal stem cell; BDNF, brain-derived neurotrophic factor; CNTF, ciliary neurotrophic factor; EGF, epidermal growth factor; GDNF, glial cell line-derived neurotrophic factor; NGF, nerve growth factor; NT-3, neurotrophin-3; NT-4, neurotrophin-4; PGRN, progranulin; TGF-β, transforming growth factor beta.

**Table 2:** Involvement of neurotrophic factors in cerebellar-related neurodevelopmental disorders.

Neurotrophic factors	Cerebellar-associated neurodevelopmental disorder	References
BDNF	ASD	Sadakata and Furuichi (2009), Sadakata et al. (2014), and Alò et al. (2021)
	MDD	Yang et al. (2017)
	BPD	Soontornniyomkij et al. (2011) and Yang et al. (2017)
	Schizophrenia	Yang et al. (2017)
	FA	Katsu-Jiménez et al. (2016)
	SCA	Cook et al. (2022) and Mellesmoen et al. (2019)
CNTF	GLD	Lin et al. (2015)
Eprins	MB	Bhatia et al. (2015)
EGF	MB	Schönholzer et al. (2020) and Neve et al. (2017)
GDNF	ADHD	Bilgiç et al. (2017) and Shim et al. (2015)
	Schizophrenia	Tunca et al. (2015)
	CMD	Sakuma et al. (2002)
NGF	ADHD	Bilgiç et al. (2017), Clemow et al. (2000), Guney et al. (2014), Syed et al. (2007), and Tiveron et al. (2013)
	MS	Damarjian et al. (2004)
Neuregulins	MB	Di Marcotullio et al. (2006) and Gilbertson et al. (1998)
	Schizophrenia	Kircher et al. (2009), Nickl-Jockschat et al. (2014), Schmitt et al. (2010), Yeganeh-Doost et al. (2011), and Barros et al. (2009)
NT-3	ASD	Sajdel-Sulkowska et al. (2009), Sajdel-Sulkowska et al. (2011), and Sadakata et al. (2014)
NT-4	CMD	Sakuma et al. (2002)
PGRN	ASD	Matsuwaki et al. (2015), Uesaka et al. (2018), and Wang et al. (2022)
TGF-b	MB	Marino (2005), Roussel and Hatten (2011), Santhana Kumar et al. (2018), and Aref et al. (2013)
	ASD	Ferretti and Hollander (2015) and Xu et al. (2017)
	CA	Cook et al. (2022) and Mellesmoen et al. (2019)

MB, medulloblastoma; ASD, autism spectrum disorder; ADHD, attention deficit hyperactivity disorder; MDD, major depressive disorder; BPD, bipolar disorder; MS, multiple sclerosis; EAE, experimental autoimmune encephalomyelitis; CMD, congenital muscular dystrophy; FA, Friedreich’s ataxia; CA, cerebellar ataxia; SCA, spinocerebellar ataxia; GLD, globoid cell leukodystrophy; BDNF, brain-derived neurotrophic factor; CNTF, ciliary neurotrophic factor; EGF, epidermal growth factor; GDNF, glial cell line-derived neurotrophic factor; NGF, nerve growth factor; NT-3, neurotrophin-3; NT-4, neurotrophin-4; PGRN, progranulin; TGF-β, transforming growth factor beta.

## BDNF

BDNF is abundantly expressed within the developing cerebellum, both embryonically and postnatally in humans and rodents (Menshanov et al., 2015; Camuso et al., 2022). While produced in both CGNs and PCs, BDNF is mostly expressed in the axons of mature CGNs of the IGL, mossy fibers (MFs), and the deep cerebellar nuclei (DCN) (Schwartz et al., 1997; Rico et al., 2002). The release of BDNF from CGN axonal terminals is facilitated by calcium-dependent activator protein for secretion 2 (CAPS2), which is a granule-associated protein (Sadakata et al., 2007; Kokubo et al., 2009; Shinoda et al., 2019). BDNF binds TrkB on both postsynaptic PCs and presynaptic CGNs, thereby leading to Trk signaling in both a paracrine and autocrine manner, respectively (Lindholm et al., 1997). Both BDNF and its immature form, proBDNF can act as a mitogenic and chemotactic factor in cerebellar development. While BDNF exerts cell survival effects through TrkB activation, proBDNF is known to be a proapoptotic mediator through activation of p75^NTR^, resulting in axon pruning and cell death (Glass et al., 1991; Ghosh et al., 1994; Singh et al., 2008).

As BDNF and TrkB are expressed in cerebellar PCs, they play a role in PC dendritogenesis and spine formation both *in vitro* and *in vivo* (Schwartz et al., 1997; Shimada et al., 1998; Yamashita et al., 2011). Lärkfors et al. (1996) found that survival of PCs increases after *in vitro* treatment with BDNF. Additionally, Morrison and Mason (1998) found that BDNF improves PC survival in isolated cultures, but decreases when co-cultured with CGNs, indicating that the neurotrophic action is context- and activity-dependent. However, recent findings suggest that BDNF does not exert a survival effect on naïve PCs *in vivo* but promotes survival in damaged PCs (Rakotomamonjy and Goumari, 2019). These data are supported by several other findings which show that BDNF does not affect survival in other neuronal populations such as cortical, hippocampal, and striatal neurons (Gorski et al., 2003; Baquet et al., 2004; Rauskolb et al., 2010).

In murine CGN cultures, BDNF–TrkB signaling promotes neurite extension and survival of differentiated mature CGNs (Gao et al., 1995; Nonomura et al., 1996; Tanaka et al., 2000). Accordingly, BDNF not only has pro-survival but also anti-apoptotic capacities in CGNs that are cultured in either serum-free media, low K^+^ media, or media with high glutamate concentrations (Lindholm et al., 1993; Kubo et al., 1995; Zirrgiebel et al., 1995; Nonomura et al., 1996; Shimoke et al., 1997; Skaper et al., 1998; Tong and Perez-Polo, 1998; Bulleit and Hsieh, 2000; Leeds et al., 2005; Sanchez-Perez et al., 2005; Ortega et al., 2010). ProBDNF, on the other hand, does not exert a pro-survival effect on CGNs. Instead, proBDNF binds to p75^NTR^, which in turn leads to the activation of the JNK signaling pathway and cell death (Koshimizu et al., 2010). Both the pro and mature form of BDNF affect the migration of CGNs *in vivo*. While endogenous BDNF promotes GCPs to exit the cell cycle and initiate migration in an autocrine manner, proBDNF acts as a negative regulator, an effect which is mediated by binding p75^NTR^ and its co-receptor sortilin (Borghesani et al., 2002; Zhou et al., 2007; Kokubo et al., 2009).

Synaptogenesis is a crucial developmental step that promotes normal brain function. Improper synapse formation is, therefore, associated with neuronal dysfunction. BDNF–TrkB signaling is involved in correct circuit wiring, synaptogenesis, and establishing a balance between inhibitory and excitatory synapses within the cerebellar system (Minichiello, 1996; Schwartz et al., 1997; Carter et al., 2002; Rico et al., 2002; Bosman et al., 2006; Shinoda et al., 2019). For instance, BDNF secreted by both excitatory MFs and CGNs aids in inhibitory synaptogenesis by regulating gephyrin, a postsynaptic scaffolding protein, clustering on CGN and PC dendrites, respectively, via the PLCy calcium-dependent and the PI3K-Akt signaling pathway (Chen et al., 2016). This is consistent with reports that BDNF–TrkB signaling promote gamma amino butyric acid (GABA)ergic synaptogenesis (Bao et al., 1999; Seil, 1999; Seil and Drake-Baumann, 2000). Moreover, BDNF contributes to the development of the two major afferent systems in the cerebellar cortex. Rabacchi et al. (1999) found that CGN-derived BDNF acts in a retrograde manner to promote the growth and maturation of innervating basilar pontine MFs. In addition, BDNF from PCs acts retrogradely on TrkB located within climbing fibers (CF) in facilitating late-phase CF synapse elimination from PC soma (Bosman et al., 2006; Choo et al., 2017). Secretion of BDNF from PCs is most likely triggered following metabotropic glutamate receptor (mGluR1) activation by parallel fiber (PF) signal transduction, the latter being a key player in CF synapse elimination (Kano et al., 1997; Ichise et al., 2000). The maturation of the cerebellar circuitry requires *de novo* synthesis of BDNF followed by activation of the TrkB-MAPK signaling pathway and phosphorylation of transcription factor ETS translocation variant 1 (Etv1) which upregulates the expression of several maturation genes with a role in dendritic development and functional synaptic assembly of the cerebellar circuit (Abe et al., 2012). Activation of Etv1 is also necessary for CaMKK2/CaMKIV-dependent phosphorylation of cAMP response element-binding protein (CREB) which drives BDNF autoregulation (Kokubo et al., 2009; Ding et al., 2018). Together, these findings demonstrate the importance of BDNF in cerebellar postnatal development as an imbalance in BDNF expression or signaling results in altered cerebellar architecture and functionality, leading to several cerebellar-related neurodevelopmental disorders which are discussed in the paragraph below.

### BDNF in cerebellar-related neurodevelopmental disorders

Impairment in BDNF signaling within the cerebellum has been implicated in several cerebellar-related disorders. Ataxia is a group of neurological disorders mainly characterized by a lack of voluntary movement coordination (Schmahmann, 2004). Post-mortem studies of patients with spinocerebellar ataxia type 6 (SCA6) show reduced expression of BDNF which was also revealed in a SCA6 mouse model, as well as a spinocerebellar ataxia type 1 (SCA1) mouse model. Both mice models display PC pathology, abnormal firing rates and changes in motor behavior. Extrinsic BDNF delivery and subsequent activation of the TrkB-Akt signaling pathway improves the PC firing rate and delays the onset of the observed motor deficits (Mellesmoen et al., 2019; Cook et al., 2022).

Friedreich’s ataxia (FA) is a predominantly neurodegenerative disease caused by recessive mutations that produce a deficiency of frataxin (FXN). FXN triggers apoptosis in CGNs, pathological changes in PCs as well as loss of motor coordination. In primary granule cultures of FXN-deficient mice, it was evidenced that BDNF can be used as a therapeutic agent that effectively prevents CGN apoptosis and PC pathogenesis (Katsu-Jiménez et al., 2016). However, this remains to be tested *in vivo*.

Furthermore, expression levels of BDNF, proBDNF, and its intrinsic receptor, TrkB, are reduced in the cerebella of patients with neuropsychiatric disorders, including schizophrenia, bipolar disorder (BPD), and major depressive disorder (MDD) as well as in a rodent model for ASD (Soontornniyomkij et al., 2011; Yang et al., 2017; Alò et al., 2021). Additionally, CAPS2-deficient mice that show reduced secretion of BDNF from CGNs exhibit developmental deficits around the cerebellar vermis, such as increased CGN apoptosis and impaired PC dendritogenesis. This leads to poor circuit connectivity and failed paired-pulse facilitation at PF-PC synapses. This is in line with changes observed in ASD patients who display cellular disturbances, as well as hypoplasia around the vermis (Sadakata and Furuichi, 2009; Sadakata et al., 2014).

## CNTF

CNTF is a cytokine that has a multifunctional role in CNS development, for instance in neurite outgrowth and neuronal survival, as well as after injury (Oyesiku and Wigston, 1996). The expression of CNTF in cerebellum is relatively low during early postnatal weeks, however, it increases significantly during adulthood (Ohta et al., 1996). Due to this low expression of CNTF in the developing cerebellum, there are limited studies on the effects of CNTF on cerebellar cells and only reports on the role of CNTF in cultured cerebellar cells. Although the expression is low, it is not absent, suggesting that CNTF might play a role during development and, therefore, warrant further studies.

One report showed that CNTF improves PC and other cerebellar GABAergic neuron survival *in vitro* as showcased in rat primary PC cultures (Lärkfors et al., 1994). Furthermore, CNTF exercises a neuroprotective effect in immature cerebellar granule cultures that are maintained in physiological low concentrations of potassium *in vitro*, which under normal circumstances, leads to apoptosis. CNTF can also prolong their survival in such non-depolarizing conditions (de Luca et al., 1996b). However, the physiological relevance of CNTF in immature CGNs remains unclear. On the other hand, CNTF might act as a differentiation factor during development. The postnatal cerebellum contains neuronal stem cells (NSCs) which derive from the white matter and CNTF has been shown to facilitate NSCs differentiation into astrocytes (Okano-Uchida et al., 2013). However, it remains unclear whether CNTF is responsible for their differentiation *in vivo*.

### CNTF in cerebellar-related neurodevelopmental disorders

Globoid cell leukodystrophy (GLD) is a lysosomal storage disease that is characterized by demyelination and astrogliosis. Such neuropathy leads to neurobehavioral changes, including cerebellar ataxia. In a murine model of GLD, cerebellar neurons as well as Bergmann glia undergo degeneration, an effect which is accompanied by the altered expression of several neurotrophic factors. For example, CNTF expression is markedly increased in GLD cerebella, which could possibly mitigate remyelination of demyelinated neurons (Lin et al., 2015).

## Ephrins

Ephrins are membrane-bound proteins that are expressed in many regions of the developing brain. They consist of two subclasses, the A-type (ephrin-A1-5) and the B-type (ephrin-B1-3) and bind to their respective tyrosine kinase receptors, namely the Eph receptors which are mainly type-specific and consist of two subfamilies, EphA and EphB (Chang et al., 1997). Their signaling is bidirectional, meaning it occurs via both phosphorylation of intracellular proteins via the Eph receptors or by intracellular signal transmission via the ephrin ligands itself upon receptor binding, a process known as reverse signaling (Rodger et al., 2012). In the chicken cerebellum, ephrin-A4 and ephrin-A5 are expressed at the earliest during embryonic stages, followed by expression of ephrin-A2 and ephrin-A3. Expression of ephrin-B1 and ephrin-B2, however, is mostly found during postnatal stages in migrating CGNs (Karam et al., 2000). Ephrin-B1 is expressed in both CGNs and PCs, while expression of its receptor, EphB, is mainly constricted to CGNs during postnatal development (Moreno-Flores et al., 2002).

The family of ephrins is involved in the formation and maintenance of PC compartments (Karam et al., 2000). For example, ephrin/Eph signaling regulates both the type and density of spines on PCs, a process which is required for defining either CFs or PFs that innervate different parts of PC dendrites. As a result, dendritogenesis on PCs is subject to competition between these fibers; CFs occupy the more proximal dendrites of PCs by suppressing the formation of smaller spines that are typically associated with PFs. Instead, CFs make room for a few larger spines to form contact with PCs. In an *in vitro* cerebellar model, it was shown that the ephrin/Eph signaling pathway affects the more proximal dendrites on PCs by inactivating integrin downstream signaling (Heintz et al., 2016). Additionally, ephrins and their receptors effectively function as PC axon guidance and growth molecules in a spatiotemporal manner (Saywell et al., 2014). One study found that both ephrin-A2 and ephrin-A5 by binding to their respective receptor control PC-MF communication during circuit formation. This is necessary for the proper patterning of mossy fiber afferents into discrete zones located within the granule layer (Lackey and Sillitoe, 2020).

While the different types of ephrin-A are crucial for PC development, ephrin-Bs regulate CGN development. For example, ephrin-B1 facilitates CGN survival, migration, dendritogenesis, as well as axonal extension (Karam et al., 2000; Sentürk et al., 2011). It also mitigates the expression of certain cell adhesion and microtubule-associated proteins which are necessary for axonal extension and guidance, as well as dendritogenesis of CGNs (Moreno-Flores et al., 2002; Wang et al., 2007). Both ephrin-B2 and its receptor EphB2 are strongly expressed in the EGL at postnatal day 3 in mice, a timepoint prior to the initiation of postmitotic CGN migration. Their concerted action is thought to inhibit the effect of certain chemokines which control the migration GCPs, consequently leading to the initiation of migration (Yacubova and Komuro, 2002). On a macroscopic scale, the ephrins and Eph receptors are thought to play an important role in cerebellar foliation as they have the ability to demarcate the cerebellar anlage (Rogers et al., 1999; Karam et al., 2000).

### Ephrins in cerebellar-related neurodevelopmental disorders

Ephrins have also been implicated in developmental disorders, in particular schizophrenia and medulloblastoma. The drug olanzapine is effective in treating schizophrenia, but its precise mechanism remains unclear. One study found that olanzapine treatment may regulate the DNA methylation of certain genes in the cerebellum, including the ephrin/Eph receptor family. This family plays a crucial role in axon guidance during development and synaptic plasticity in adulthood, including long-term potentiation, which has been linked to psychosis. Therefore, the epigenetic changes in these genes may account for the therapeutic effects of olanzapine observed in a rat model of schizophrenia (Melka et al., 2014).

Medulloblastoma (MB) is an aggressive tumor that arises from GCPs in the cerebellum. Proper formation and migration of these precursors require ephrin-A5 and its receptors. In a mouse model of MB, it was found that deletion of ephrin-A5 inhibits tumor growth, providing a platform for development of ephrin-based pharmacological interventions of medulloblastoma (Bhatia et al., 2015).

## EGF

EGF is part of the large EGF superfamily which also contains the transforming growth factor alpha (TGF-α) and the neuregulins (Wieduwilt and Moasser, 2008). It binds the epidermal growth factor receptor (EGFR, also called ErbB1) or receptor homologs ErbB2, ErbB3, or ErbB4 (Wieduwilt and Moasser, 2008). EGF and its receptor, EGFR, are expressed during all stages of life depending on the cell type, however, during postnatal development, they can be found within the CGNs, PCs and astrocytes of the cerebellar cortex as well as the DCN (Gómez-Pinilla et al., 1988; Seroogy et al., 1995; Scalabrino, 2022). EGF binding to EGFR results in activation of several signaling pathways including the PLC, PI3-K, and the Ras-MAPK signaling pathway (Wong and Guillaud, 2004).

EGF has the ability to support neuronal growth and survival in cultured rodent CGNs (Morrison et al., 1988; Abe et al., 1991, 1992; Yamada et al., 1997). This indicates that EGF can act as a neurotrophic factor that promotes the elongation and maintenance of neurites in cerebellar neurons which is most likely achieved through the activation of protein kinases (Morrison et al., 1988; Abe et al., 1991, 1992; Yamada et al., 1997). Moreover, EGF effectively reduces glutamate-associated apoptosis in primary CGN cultures and has thus a neuroprotective effect against glutamate-induced neurotoxicity (Abe and Saito, 1992; Gunn-Moore and Tavaré, 1998). In addition, it also enhances survival of serum-deprived cerebellar cultures (Leutz and Schachner, 1981). Concomitant to its function in CGN survival and maturation, EGF has been suggested to be involved in CGN migration, largely due to expression of EGFR found in premigratory post-mitotic CGNs and its function in facilitating Bergmann glia elongation (Carrasco et al., 2003; Martinez et al., 2011). NSCs which derive from the cerebellar white matter require EGF to keep their proliferative ability (Okano-Uchida et al., 2013). Once these NSCs differentiate into astrocytes they express EGFR. It has been reported that EGF-EGFR signaling in these astrocytes stimulates DNA synthesis increasing their proliferation (Leutz and Schachner, 1981).

### EGF cerebellar-related neurodevelopmental disorders

Similar to ephrins, EGF signaling is involved in MB as it rapidly increases nuclear activation of the ERK1/2-MAPK pathway in MB cells which speeds the invasion of these cells (Schönholzer et al., 2020). Neve et al. (2017) found that in organotypic cerebellar slices, EGF effectively enhances tumor growth and infiltration, indicating its tumor progressing capabilities.

## GDNF

GDNF is expressed in several different types of neurons, including PCs, and plays a crucial role in regulating various processes in the developing nervous system, such as neuron survival, cell migration, axon growth, and synapse formation (Mount et al., 1995; McAlhany et al., 1997, 1999; Paratcha and Ledda, 2008). It acts in a paracrine manner by pairing with the GDNF family receptor α1 (GFRα1) (Treanor et al., 1996). The GDNF/GFRa1 complex subsequently binds with the “rearranged during transfection” (RET) tyrosine kinase receptor or the neural cell adhesion molecule (NCAM), though with lower affinity to activate either the MAPK or P13K–Akt pathway (Treanor et al., 1996; Trupp et al., 1996; Paratcha and Ledda, 2008; Sergaki and Ibáñez, 2017).

GDNF, expressed by PCs, has been shown to be crucial for the survival of molecular layer interneurons (MLIs) during postnatal cerebellar development (Sergaki and Ibáñez, 2017). It binds the GFRa1-RET receptor complex on MLIs in a cell-autonomous manner to stimulate IN survival. The absence of either receptor leads to the loss of MLIs, decreased GABAergic inputs to PC dendrites, and an increase in PC firing rate, subsequently resulting in compromised motor learning as well as eyeblink conditioning (Sergaki and Ibáñez, 2017). Additionally, GDNF has neurotrophic capacities in cultured PCs as it aids spine formation and thickening of the dendritic tree as well as in CGNs as it increases survival of these neurons (Mount et al., 1995; Subramaniam et al., 2008).

### GDNF in cerebellar-related neurodevelopmental disorders

GDNF has been implicated in several cerebellar-related neurodevelopmental disorders including schizophrenia and ADHD. For example, serum of schizophrenia patients shows reduced levels of GDNF compared to healthy controls (Tunca et al., 2015). Conversely to schizophrenia, plasma levels of GDNF in children with ADHD are markedly increased (Shim et al., 2015; Bilgiç et al., 2017). Although the levels of GDNF are altered in both schizophrenia and ADHD children, both studies measured circulation levels of GDNF suggesting that this alteration might not be restricted to the cerebellum but a global nervous system impairment.

Furthermore, GDNF might also play a role in congenital muscular dystrophy (CMD). CMD encompasses a group of genetic muscle diseases, characterized by muscle weakness, hypotonia, and muscle atrophy and often accompanied by respiratory complications as well as intellectual disability (Bertini et al., 2011). In a mouse model of CMD, it was found that GDNF expression was markedly enhanced in both PCs and CGNs (Sakuma et al., 2002). Since GDNF is a potent PC survival agent, such elevated expression levels may be the result of a compensatory mechanism due to PC degeneration. Conversely, exuberant levels of GDNF may be neurotoxic to the PCs and contribute to cerebellar degeneration.

## NGF

NGF expression has been shown in many brain regions, most prominently in the cerebellum (Shelton and Reichardt, 1986). Studies from our and other groups have reported expression of NGF in CGNs (Matsui et al., 1990; Cohen-Cory et al., 1993; Kisiswa et al., 2018). NGF can bind two distinct receptors, TrkA and p75^NTR^, where activation of TrkA induces multiple signaling pathways such as PI3K-Akt and MAPK that regulate cellular survival, differentiation, and neurite outgrowth (Crowder and Freeman, 1998; Kaplan and Miller, 2000). The proform of NGF, proNGF is thought to induce apoptosis when bound to p75^NTR^ in the presence of sortilin, but also acts as a growth factor and induces neurite outgrowth when bound to TrkA in the absence of p75^NTR^ (Nykjaer et al., 2004; Buttigieg et al., 2007). In the cerebellum, p75^NTR^ is expressed in PCs and CGNs (Pioro and Claudio Cuello, 1988; Kisiswa et al., 2018). TrkA, on the other hand, is not expressed in the healthy cerebellum, indicating that NGF signals through p75^NTR^ to exert its neurotrophic capacities.

NGF has been implicated as a differentiation and a survival factor for PCs *in vitro* in the presence of BDNF/TrkB signaling support (Cohen-Cory et al., 1991; Legrand and Clos, 1991; Mount et al., 1998; Florez-McClure et al., 2004). Not only in PCs, NGF also promotes the proliferation of immature granule cells and the postmitotic survival of newly differentiated CGNs (Legrand and Clos, 1991; Muller et al., 1994). The *in vitro* and *in vivo* survival effect of NGF on CGNs is RIP2-dependent and leads to an increase in NF-κB activity (Khursigara et al., 2001). In the absence of RIP2, however, NGF can induce JNK-dependent apoptosis (Kisiswa et al., 2018). The ability of the NGF-p75^NTR^ complex to induce cell death in CGNs is normally suppressed or masked by concurrent activation of NF-kB signaling (Vicario et al., 2015). proNGF, on the other hand, displays proapoptotic activity through the increase of c-Jun phosphorylation via the JNK-dependent pathway. Therefore, NGF and proNGF function in an antagonistic manner, and their balance is key deterministic in CGN survival during postnatal cerebellar development. It is worth noting that immature granule neuron-derived NGF can induce an increase in intracellular calcium through the ryanodine receptor, which is followed by a rapid release of glutamate via the p75^NTR^-dependent pathway. Such release of glutamate from PF terminals is important for the strengthening of PF-PC synapses (Numakawa et al., 2003).

### NGF in cerebellar-related neurodevelopmental disorders

ADHD has recently been associated with the cerebellum, although the degree of cerebellar contribution to ADHD pathophysiology requires further studies (Mackie et al., 2007; Sathyanesan et al., 2019). Nevertheless, the levels of NGF in ADHD animal models and in children with ADHD is significantly increased in blood samples (Clemow et al., 2000; Syed et al., 2007; Tiveron et al., 2013; Guney et al., 2014; Bilgiç et al., 2017). However, like GDNF, the current data indicate that the alteration of NGF in these patients is a global effect and not cerebellar-specific. Considering the beneficial effect of NGF on cerebellar neurons, we speculate that the increase of NGF could be a compensatory mechanism to protect the cerebellum from impairment caused by ADHD.

Patients with multiple sclerosis (MS) and animal models of experimental autoimmune encephalomyelitis (EAE) often display cerebellar ataxia. This pathophysiological phenomenon is partly caused by abnormal PC firing as a result of an imbalance in sodium channel expression. NGF acting via p75^NTR^ has the ability to modulate the expression of sodium channel Nav1.8 in PCs and, therefore, contribute to the regular PC firing rate. In a murine EAE model, it was revealed that levels of NGF and p75^NTR^ are increased which leads to the upregulation of Nav1.8 channels (Damarjian et al., 2004).

## Neuregulins

Neuregulins are a group of trophic factors that are part of the large EGF superfamily and have an essential role in both the developing brain and during synaptic plasticity in the adult brain (Wong and Guillaud, 2004). The neuregulins which are composed of neuregulin-1 (or heregulin), neuregulin-2, neuregulin-3, and neuregulin-4 interact with the ErbB family of receptors (Chang et al., 1997). Both the neuregulins and their receptors are expressed in a spatiotemporal manner within the developing cerebellum. Neuregulins and ErbB2, ErbB3 and ErbB4 are all expressed in CGNs while ErbB4 is also expressed in radial glial cells such as the Bergmann glia (Rio et al., 1997; Ozaki et al., 1998; Rahman et al., 2019). In the maturing cerebellum, neuregulins are concentrated in glutamatergic MFs that innervate CGNs located in the IGL (Ozaki et al., 1997). Several studies found that in murine primary cultures of CGN, neuregulin-1 signals through the ErbB4 receptor to regulate its interaction with PSD95 which is crucial for CGN differentiation. The C-terminal part of the ErbB4 receptor associates with PSD95 leading to the assembly of the nitric oxide synthase (NOS)-1 complex, a process which is thought to be mediated via the MAPK pathway (Krainock and Murphy, 2001a,b; Murphy and Bielby-Clarke, 2008). Neuregulin-1 also promotes differentiation and morphogenesis of Bergmann glia which in turn, enhances migration of CGNs (Rio et al., 1997; Yacubova and Komuro, 2002; Buffo and Rossi, 2013).

One *in vivo* study found that neuregulin–ErbB signaling initiates dendritogenesis and maturation of postsynaptic compartments in the developing murine cerebellum (Rieff and Corfas, 2006). Neuregulins have the ability to serve as cell adhesion molecules on CGNs for synaptic recognition of MFs, leading to the formation of the cerebellar MF system (Ozaki et al., 2000; Ozaki, 2001). These growth factors are not only essential for glutamatergic circuit wiring as they have also been ascribed a role in the GABA system as well. More specifically, neuregulin-1 through activation of the ErbB4 receptor tyrosine kinase effectively induces expression of the GABA_A_ receptor β2 subunit via the MAPK, PI3K, and the cyclin-dependent kinase-5 (cdk5) pathway in primary CGN cultures (Xie et al., 2004). Activation of cdk5 leads to recruitment of PSD95 which in turn facilitates the effects of neuregulin through its interaction with ErbB4. As a result, this mechanism functions as a positive feedback system to neuregulin signaling and consequent expression of the GABA_A_ β2 subunit (Xie et al., 2007). Extrinsic delivery of neuregulin causes an increase in the GABA_A_ receptor β2 subunit expression of CGN cultures *in vitro* which is paralleled by an increase in functional GABA_A_ receptors (Rieff et al., 1999).

It has been reported that neuregulin can mediate synaptogenesis via two distinctive mechanisms. First, the soluble form of neuregulin can be proteolytically cleaved from the membrane-associated form as a result of protein kinase activation. This soluble form is then able to transsynaptically and in a paracrine manner act as a neurotrophic factor and regulate the expression of the NMDA receptor subunit NR2C (Ozaki et al., 2000). The soluble form of neuregulin-1 can be shedded in a frequency-dependent manner due to electrical stimulation in both CGN and pontine nucleus neurons that form MF afferents and synapse onto CGN. Such cleaved neuregulin-1 is thus important for synaptic transmission across MF-CGN synapses (Ozaki et al., 2004). Second, the membrane-anchored form in both CGNs and MF terminals can serve as a cell-recognition molecule to stimulate MF-CGN synapse formation (Ozaki et al., 2000). However, there is some debate on neuregulins’ role in synaptogenesis as one study found that neuregulin/ErbB signaling to CGNs is dispensable for the normal development of their synaptic inputs as compared to previous *in vitro* experiments (Gajendran et al., 2009). Nevertheless, expression of NR2C is specifically induced during synaptogenesis of CGNs within the IGL, leading to dramatic changes in the NMDA receptor composition during development. As neuregulins are expressed in the MFs that innervate CGNs located in the IGL, one study found that cultured cerebellar slices stimulated with a neuregulin isoform dramatically increase the expression of NR2C messenger RNAs. This mechanism is mediated by the binding of neuregulins onto its receptors ErbB2 and ErbB4 located on CGNs. In conclusion, cell-autonomous signaling of NRG1/ErbB can modulate both glutamatergic and GABAergic neurotransmitter receptor composition during development and regulate synaptic plasticity (Ozaki et al., 1997; Fenster et al., 2012).

### Neuregulins in cerebellar-related neurodevelopmental disorders

Multiple studies have suggested that the neuregulin-1 gene (NRG1) serves as an important risk gene for schizophrenia that is thought to be characterized by deficits in glutamatergic neurotransmission (Kircher et al., 2009; Nickl-Jockschat et al., 2014). In patients with schizophrenia, it was found that gene expression of the NMDA receptor subunit 2D (NMDAR2D) was significantly increased in the cerebellum, which results in a hyperexcitability of the NMDA receptor, and which may be a secondary upregulation due to a dysfunctional receptor. In patients with the NRG1 risk variant, they found that expression of the NMDAR2C subunit was significantly reduced which could lead to hypofunctionality of the NMDA receptor, that in turn may lead to dysfunction of the GABA system (Schmitt et al., 2010). Accordingly, from post-mortem studies, there is accumulating evidence that GABAergic signaling is decreased in the cerebellum of schizophrenia patients (Yeganeh-Doost et al., 2011). Not only NRG1, but also its receptors, ErbB2 and ErbB4 are candidate susceptibility genes for schizophrenia. While deletion of ErbB2 and ErbB4 does not affect brain anatomy on a macroscopic scale, it can lead to impaired spine maturation as well impaired interactions with postsynaptic scaffold proteins, such as PSD95, with glutamate receptors which in turn leads to behavioral abnormalities (Barros et al., 2009).

MB is associated with decreased activity of the mitogen sonic hedghehog (shh). Under normal conditions, shh downregulates the expression of ErbB4, while in MB subsets, there is accumulation of both ErbB2 and ErbB4. This leads to both anti-apoptotic and loss of cell growth arrest signaling in neuronal progenitors of the cerebellum (Gilbertson et al., 1998; Di Marcotullio et al., 2006).

## NT-3

In the developing cerebellum, NT-3 and its high-affinity tyrosine kinase receptor TrkC are expressed by both the differentiated CGNs of the IGL and their precursors in the EGL as well as in PCs (Neveu and Arenas, 1996; Doughty et al., 1998). In the rodent cerebellum, levels of NT-3 markedly decrease after the first 10 postnatal days (Katoh-Semba et al., 2000).

Formation of the PC arbor and competitive dendritogenesis in the mouse cerebellum is regulated by NT-3, expressed in CGNs, a process which is TrkC-dependent (Neveu and Arenas, 1996; Joo et al., 2014; Tepper et al., 2020). In cultured PCs, NT-3 effectively increases cell numbers via the PKC-dependent pathway while enhancing their survival and phenotypic differentiation (Lärkfors et al., 1996; Mount et al., 1998). Concomitant to BDNF, CAPS2-mediated NT-3 release is known to be involved in the development and maturation of synapses and the balance between inhibitory and excitatory synapses (Sadakata et al., 2014; Shinoda et al., 2019). In addition, NT-3 promotes initial olivary axonal outgrowth to the cerebellar cortex and early CF synaptogenesis onto PCs (Sherrard and Bower, 2002).

NT-3 also has an important role in CGN development. In the murine cerebellum, NT-3 promotes the differentiation of premigratory granule cells, accelerating the cell cycle exit (Neveu and Arenas, 1996; Doughty et al., 1998; Takumi et al., 2005). It does this via either direct autocrine signaling, indirect via PCs, or by a combination of both and synergistically to BDNF (Minichiello, 1996). More recently, it was found that proNT-3, and not mature NT-3, affects GCP proliferation and differentiation. ProNT-3 binds to p75^NTR^ and the co-receptor SorCS2, which is a member of the sortilin receptor family, to antagonize the shh-induced proliferation of GCPs and initiate cell cycle exit (Zanin et al., 2016, 2019; Zanin and Friedman, 2022). NT-3, on the other hand, aids the migration of newly differentiated GCPs from the EGL *in vivo*, an effect which is antagonized by p75^NTR^ (Neveu and Arenas, 1996). Since GCPs express p75^NTR^ during their proliferative but not their migratory state, p75^NTR^ effectively prevents GCPs from migrating by maintaining elevated levels of active RhoA, a member of the Rho-GTPase family that plays a role in neuronal migration (Zanin and Friedman, 2022). Once CGPs have differentiated to CGNs and completed proliferation, NT-3 provides maturation support to these neurons (Segal et al., 1992). *In vitro*, NT-3 is known to support the survival of mature CGNs via TrkC (Bates et al., 1999; Katoh-Semba et al., 2000; Joo et al., 2014) and provide neuroprotective capacities in low K^+^ cultured CGNs (Kubo et al., 1995; Shimoke et al., 1997; Bates et al., 1999; Katoh-Semba et al., 2000; Joo et al., 2014).

### NT-3 in cerebellar-related neurodevelopmental disorders

Abnormalities in the expression of NT-3 have been associated with autism spectrum disorders. Exorbitant levels of NT-3 affect normal axonal targeting and synapse formation, and result in a decrease in PC numbers, all of which are effects seen in ASD pathology (Sajdel-Sulkowska et al., 2009, 2011). CAPS2 is essential for the release of NT-3 from CGNs, however, in patients with ASD, an alternative splice variant of CAPS2 that lacks exon 3, namely dex3, alters the release of NT-3. In a representative mouse model, NT-3 is markedly reduced in the axons of CGNs, an effect that results in reduced PC arborization and GCP proliferation. This leads to both a smaller vermal volume, as well as impaired paired-pulse facilitation at PF-PC synapses which is in line with autistic phenotypes (Sadakata et al., 2014).

## NT-4

NT-4 has similar properties to BDNF within the developing cerebellum, although its expression levels peak higher during the first postnatal week (Proenca et al., 2016). NT-4 signals predominantly through the TrkB receptor and has a functional role in both CGN and PC maturation as well as survival and inhibitory synaptogenesis (Proenca et al., 2016). On the one hand, NT-4 improves the survival of isolated PCs (Morrison et al., 1988) as well as their phenotypic differentiation *in vitro* (Morrison et al., 1988; Lärkfors et al., 1996). In CGNs, on the other hand, NT-4 promotes survival as well as neurite extension and dendritic arborization in a similar manner to BDNF, via the TrkB-dependent pathway (Gao et al., 1995). In murine CGN cultures, NT-4 has a cytoprotective capacity and can circumvent glutamate-induced oxidative death via activation of the PI3K or MAPK pathway (Kubo et al., 1995; Shimoke et al., 1997; Skaper et al., 1998). NT-4 has similar capacities to BDNF and NT-3 in promoting correct circuit wiring of the cerebellar cortex and, concomitant to BDNF, NT-4 aids inhibitory synaptogenesis (Seil, 1999). Additionally, NT-4 promotes initial olivary axonal growth to the cerebellar plate and CF synaptogenesis as well as axonal outgrowth and survival of pontocerebellar MF neurons (Rabacchi et al., 1999; Sherrard and Bower, 2002).

### NT-4 in cerebellar-related neurodevelopmental disorders

In a murine model for CMD, expression of NT-4 is markedly reduced in the cerebellum, in the spinal cord, and hindlimb muscles. Such a marked decrease may contribute to the progressive degeneration of muscle fibers and, due to its role in CGN and PC survival, cerebellar hypoplasia (Sakuma et al., 2002).

## PGRN

PGRN is the precursor protein for granulin, expressed in both the periphery and central nervous system (Townley et al., 2018). The propeptide is mainly delivered into lysosomes as it plays a role in regulating protein homeostasis via the lysosomal pathway (Paushter et al., 2018). It has also been hypothesized to have neurotrophic capacities and function as an autocrine neuronal growth factor (Van Damme et al., 2008; Paushter et al., 2018). The expression levels of PGRN are thought to be regulated by the sortilin receptor which mediates its lysosomal endocytosis (Kao et al., 2017). However, PGRN can also exert its neurotrophic properties in a sortilin-independent manner, via prosaposin which carries PGRN into the lysosomes and regulates its expression (Zhou et al., 2015). PGRN is highly expressed in the cerebellar PCs during the late stages of postnatal development (Matsuwaki et al., 2015).

Matsuwaki et al. (2015) found that in PGRN-deficient mice, the PC dendritic density is significantly increased, possibly due to a lack of synaptic pruning, while no changes occur in the number of PCs. Taken together, this suggests that PGRN affects PC dendritogenesis but not neurogenesis and/or survival. However, in a study by Wang et al. (2022), it was found that PGRN aids neuronal survival and synaptic development via activation of the PI3K-Akt signaling pathway. In accordance with its role in synapse formation, PGRN also plays a role in defining CFs as a PC-derived regulator, by both counteracting redundant CFs and reinforcing the strongest CF inputs via the sortilin-dependent pathway. This retrograde mechanism is driven by voltage-gated calcium channels and the mGLuR1 signaling cascade (Uesaka et al., 2018).

### PGRN in cerebellar-related neurodevelopmental disorders

PGRN has previously been implicated in cerebellar-associated degenerative but also in neurodevelopmental disorders (Matsuwaki et al., 2015; Simonati and Williams, 2022). It has been shown that PGRN is essential for PC dendritogenesis and CF-PC synaptogenesis and that abnormalities in PGRN expression may lead to synaptic disturbances as well as behavioral deficits, such as impaired motor function and coordination, reduced social preference, and increased repetitive behaviors (Matsuwaki et al., 2015; Uesaka et al., 2018; Wang et al., 2022). These behavioral phenotypes are all characteristic of those seen in ASD patients. Wang et al. (2022) found that abnormal spatiotemporal expression of PGRN is related to neurodevelopmental impairments in an ASD murine model.

## TGF-β

TGF-β is a multipotent cytokine which is generally induced by acute or chronic brain injury, however, it also has cell differentiation, proliferation and apoptotic capacities during development (Dobolyi and Palkovits, 2008). Nonetheless, TGF- β does not only exert a neuroprotective function, as it can also induce neuronal and glial degeneration after injury (Wang et al., 1995; Yamashita et al., 1999). TGF-β exists as three isoforms in mammals, namely TGF-β1, TGF-β2, and TGF-β3 (Voisin et al., 2020). Under normal conditions, TGF-β is scarcely expressed within the cerebellum, however, TGF-β2 expression can be found in GCPs of the EGL and post-mitotic CGNs located in the IGL of the developing cerebellar cortex until postnatal day 10. It does remain expressed in PCs during both development and adulthood (Constam et al., 1994; Kane et al., 1996). TGF-β1 expression, on the other hand, is low during early postnatal stages but increases after postnatal day 12 and remains high until postnatal day 30 (Araujo et al., 2016). TGF- β signaling is initiated by the binding of extracellular TGF-β ligands to their respective receptors forming a complex. This complex formation allows phosphorylation of Smad proteins which then translocate to the cell nucleus to regulate the expression of multiple early target genes, including those that have a role in cell proliferation and differentiation, for example ID1-3, CDKN1A, OVOL1 and JUNB (Kowanetz et al., 2004; Zhang et al., 2016). However, their effects on cerebellar neurons are yet to be unraveled. We, therefore, propose that more studies are warranted for delineating cellular mechanisms regarding cerebellar-related neurodevelopmental disorders.

Deletion of Smad4, a critical mediator of TGF-β, results in Purkinje and GABAergic interneuron cell loss which leads to neurobehavioral deficits, including motor dysfunction (Zhou et al., 2003). Another study found that Smad2, another mediator of TGF-β signaling and highly expressed in the mouse brain during early postnatal development, is necessary for proper cerebellar foliation. Absence of Smad2 results in aberrant PC dendritic arborization and cell loss as well as other cerebellar deficits such as increased apoptosis and defect migration of CGNs which leads to motor dyscoordination (Wang et al., 2011).

TGF-β has a pro-survival and pro-growth effect on GCPs (Elvers et al., 2005). TGF-β1 effectively increases the number of glutamatergic synapses in CGN cultures, an effect which is dependent on binding to its receptor, TβRII. TGF-β1 thus mediates excitatory synapse formation in CGNs (Araujo et al., 2016). Moreover, TGF-β1 has the capacity to change the electrophysiological properties and voltage-dependent ion currents of CGNs after injury which leads to functional changes in the CNS (Ondáčová et al., 2017). However, TGF-β can also serve as a pro-apoptotic agent in low K^+^ cultured CGNs (de Luca et al., 1996a). More specifically, TGF-β1 has a neurotoxic effect on mixed neuronal and astrocytic cultures as the CGNs become dependent on astrocytes for survival. TGF-β1 acts as a cytokine and inhibits the ability of astrocytes to clear glutamate, which leads to an increase in the glutamate concentration within the media that is toxic to the CGNs and eventually decreases their survival (Brown, 1999). TGF-β2, on the other hand, differently regulates proliferation and survival of CGNs, depending on the media conditions. TGF-β2 functions as a proliferative agent in serum-treated media, while it inhibits proliferation in serum-free media, indicating that as it requires exogenous regulatory factors (Constam et al., 1994; Kane et al., 1996).

### TGF-β in cerebellar-related neurodevelopmental disorders

MB is thought to arise from disruptions in cerebellar development and growth factors, such as TGF-β, are thought to play a role in its progression (Marino, 2005; Roussel and Hatten, 2011; Santhana Kumar et al., 2018). The canonical TGF-β signaling pathway involves activation of Smad3 in a subset of GCPs that possibly represents the putative cells of origin for MB (Aref et al., 2013).

In a murine model of autism, TGF-β1 expression is significantly decreased within the cortex, hippocampus, and cerebellum. This is in line with a previous study which found reduced levels in patients with ASD (Ferretti and Hollander, 2015; Xu et al., 2017).

Cerebellar ataxia (CA) is usually accompanied by microglia-mediated neuroinflammation, yet how it contributes to cerebellar pathogenesis remains unsolved. In one study of CA model rats, it was found that exogenous administration of anti-inflammatory TGF-β1 reduces neuronal loss and microglial activation in both brain stem and cerebellum, and consequently ameliorates motor deficits as seen in CA (Cao et al., 2020). In another study of cerebellar ataxia, it was found that TGF-β1 is significantly upregulated, likely as a result of increased neuroinflammation (Jiang et al., 2015).

## Conclusion

Neurotrophic factors and their receptors exert different cellular functions, and their spatiotemporal expression is crucial for the normal development of the cerebellar cytoarchitecture. In this review, we mainly focused on the purpose of these factors during the postnatal development of cells within the cerebellar cortex. However, several are also expressed during adulthood and play a role in both short-term and long-term synaptic plasticity. Because of their multifaceted features in neuronal differentiation, survival, synaptogenesis, and circuit wiring, it is inferred that the ablation of these factors can lead to serious defects on the tissue, cellular and molecular levels. A range of neurological disorders with a cerebellar component and abnormalities in either neurotrophic factor expression and/or activity have been discussed. It is important to note that this is not restricted to motor disorders, which are known to involve the cerebellar system, but also non-motor disorders. This indicates that the cerebellum, supplementing its role in motor performance, also plays a crucial role in cognitive and emotional development. Further studies regarding neurotrophic factors and the effector downstream signaling mechanisms within the cerebellar system could illuminate whether they might serve as pharmacological agents to moderate certain disease models.

## Author contributions

All authors listed have made a substantial, direct, and intellectual contribution to the work and approved it for publication.

## Funding

This work was supported by Lundbeck Foundation grant no. R248-2017-431 and PROMEMO grant DNRF133.

## Conflict of interest

The authors declare that the research was conducted in the absence of any commercial or financial relationships that could be construed as a potential conflict of interest.

## Publisher’s note

All claims expressed in this article are solely those of the authors and do not necessarily represent those of their affiliated organizations, or those of the publisher, the editors and the reviewers. Any product that may be evaluated in this article, or claim that may be made by its manufacturer, is not guaranteed or endorsed by the publisher.

## References

[ref1] Abe H. Okazawa M. Nakanishi S. (2012). Gene regulation via excitation and Bdnf is mediated by induction and phosphorylation of the Etv1 transcription factor in cerebellar granule cells. Proc. Natl. Acad. Sci. 109, 8734–8739. doi: 10.1073/pnas.1206418109, PMID: 22586091 PMC3365191

[ref2] Abe K. Saito H. (1992). Protective effect of epidermal growth factor on glutamate neurotoxicity in cultured cerebellar neurons. Neurosci. Res. 14, 117–123. doi: 10.1016/0168-0102(92)90087-S, PMID: 1356258

[ref3] Abe K. Takayanagi M. Saito H. (1991). Basic fibroblast growth factor and epidermal growth factor promote survival of primary cultured cerebellar neurons from neonatal rats. Jpn. J. Pharmacol. 56, 113–116. doi: 10.1016/S0021-5198(19)39906-8, PMID: 1880981

[ref4] Abe K. Takayanagi M. Saito H. (1992). Neurotrophic effects of epidermal growth factor on cultured brain neurons are blocked by protein kinase inhibitors. Japan. J. Pharmacol. 59, 259–261. doi: 10.1254/jjp.59.259, PMID: 1434124

[ref5] Adamaszek M. D’agata F. Ferrucci R. Habas C. Keulen S. Kirkby K. C.. (2017). Consensus paper: cerebellum and emotion. Cerebellum 16, 552–576. doi: 10.1007/s12311-016-0815-8, PMID: 27485952

[ref6] Allen G. Müller R.-A. Courchesne E. (2004). Cerebellar function in autism: functional magnetic resonance image activation during a simple motor task. Biol. Psychiatry 56, 269–278. doi: 10.1016/j.biopsych.2004.06.005, PMID: 15312815

[ref7] Alò R. Olivito I. Fazzari G. Zizza M. Di Vito A. Avolio E.. (2021). Correlation of distinct behaviors to the modified expression of cerebral Shank1,3 and Bdnf in two autistic animal models. Behav. Brain Res. 404:113165. doi: 10.1016/j.bbr.2021.113165, PMID: 33577886

[ref8] Altman J. (1972). Postnatal development of the cerebellar cortex in the rat. Ii. Phases in the maturation of Purkinje cells and of the molecular layer. J. Comp. Neurol. 145, 399–463. doi: 10.1002/cne.901450402, PMID: 5044254

[ref9] Amore G. Spoto G. Ieni A. Vetri L. Quatrosi G. Di Rosa G.. (2021). A focus on the cerebellum: from embryogenesis to an age-related clinical perspective. Front. Syst. Neurosci. 15:646052. doi: 10.3389/fnsys.2021.646052, PMID: 33897383 PMC8062874

[ref10] Araujo A. P. B. Diniz L. P. Eller C. M. De Matos B. G. Martinez R. Gomes F. C. A. (2016). Effects of transforming growth factor Beta 1 in cerebellar development: role in synapse formation. Front. Cell. Neurosci. 10:104. doi: 10.3389/fncel.2016.00104, PMID: 27199658 PMC4846658

[ref11] Aref D. Moffatt C. J. Agnihotri S. Ramaswamy V. Dubuc A. M. Northcott P. A.. (2013). Canonical Tgf-β pathway activity is a predictor of Shh-driven medulloblastoma survival and delineates putative precursors in cerebellar development. Brain Pathol. 23, 178–191. doi: 10.1111/j.1750-3639.2012.00631.x, PMID: 22966790 PMC8029114

[ref12] Bao S. Chen L. Qiao X. Thompson R. F. (1999). Transgenic brain-derived neurotrophic factor modulates a developing cerebellar inhibitory synapse. Learn. Memory 6, 276–283. doi: 10.1101/lm.6.3.276, PMID: 10492009 PMC311309

[ref13] Baquet Z. C. Gorski J. A. Jones K. R. (2004). Early striatal dendrite deficits followed by neuron loss with advanced age in the absence of anterograde cortical brain-derived neurotrophic factor. J. Neurosci. 24, 4250–4258. doi: 10.1523/JNEUROSCI.3920-03.2004, PMID: 15115821 PMC6729276

[ref14] Barros C. S. Calabrese B. Chamero P. Roberts A. J. Korzus E. Lloyd K.. (2009). Impaired maturation of dendritic spines without disorganization of cortical cell layers in mice lacking Nrg1/ErbB signaling in the central nervous system. Proc. Natl. Acad. Sci. 106, 4507–4512. doi: 10.1073/pnas.0900355106, PMID: 19240213 PMC2657442

[ref15] Bates B. Rios M. Trumpp A. Chen C. Fan G. Bishop J. M.. (1999). Neurotrophin–3 is required for proper cerebellar development. Nat. Neurosci. 2, 115–117. doi: 10.1038/5669, PMID: 10195193

[ref16] Bertini E. D'amico A. Gualandi F. Petrini S. (2011). Congenital muscular dystrophies: a brief review. Semin. Pediatr. Neurol. 18, 277–288. doi: 10.1016/j.spen.2011.10.010, PMID: 22172424 PMC3332154

[ref17] Bhatia S. Hirsch K. Baig N. A. Rodriguez O. Timofeeva O. Kavanagh K.. (2015). Effects of altered ephrin-A5 and EphA4/EphA7 expression on tumor growth in a medulloblastoma mouse model. J. Hematol. Oncol. 8:105. doi: 10.1186/s13045-015-0202-9 26345456 PMC4561476

[ref18] Bilgiç A. Toker A. Işık Ü. Kılınç İ. (2017). Serum brain-derived neurotrophic factor, glial-derived neurotrophic factor, nerve growth factor, and neurotrophin-3 levels in children with attention-deficit/hyperactivity disorder. Eur. Child Adolesc. Psychiatry 26, 355–363. doi: 10.1007/s00787-016-0898-2, PMID: 27561780

[ref19] Bonni A. Frank D. A. Schindler C. Greenberg M. E. (1993). Characterization of a pathway for ciliary neurotrophic factor signaling to the nucleus. Science 262, 1575–1579. 7504325 10.1126/science.7504325

[ref20] Borghesani P. R. Peyrin J. M. Klein R. Rubin J. Carter A. R. Schwartz P. M.. (2002). Bdnf stimulates migration of cerebellar granule cells. Development 129, 1435–1442. doi: 10.1242/dev.129.6.1435, PMID: 11880352

[ref21] Bosman L. W. J. Hartmann J. Barski J. J. Lepier A. Noll-Hussong M. Reichardt L. F.. (2006). Requirement of TrkB for synapse elimination in developing cerebellar Purkinje cells. Brain Cell Biol. 35, 87–101. doi: 10.1007/s11068-006-9002-z 17940915 PMC3303929

[ref22] Brown D. R. (1999). Dependence of Neurones on astrocytes in a Coculture system renders Neurones sensitive to transforming growth factor \gb\1-induced glutamate toxicity. J. Neurochem. 72, 943–953. doi: 10.1046/j.1471-4159.1999.0720943.x, PMID: 10037465

[ref23] Buffo A. Rossi F. (2013). Origin, lineage and function of cerebellar glia. Prog. Neurobiol. 109, 42–63. doi: 10.1016/j.pneurobio.2013.08.001, PMID: 23981535

[ref24] Bulleit R. F. Hsieh T. (2000). Mek inhibitors block Bdnf-dependent and -independent expression of Gabaa receptor subunit mrnas in cultured mouse cerebellar granule neurons. Dev. Brain Res. 119, 1–10. doi: 10.1016/S0165-3806(99)00119-4, PMID: 10648867

[ref25] Buttigieg H. Kawaja M. D. Fahnestock M. (2007). Neurotrophic activity of prongf in vivo. Exp. Neurol. 204, 832–835. doi: 10.1016/j.expneurol.2006.12.011, PMID: 17270174

[ref26] Camuso S. La Rosa P. Fiorenza M. T. Canterini S. (2022). Pleiotropic effects of Bdnf on the cerebellum and hippocampus: implications for neurodevelopmental disorders. Neurobiol. Dis. 163:105606. doi: 10.1016/j.nbd.2021.105606, PMID: 34974125

[ref27] Cao B.-B. Zhang X.-X. Du C.-Y. Liu Z. Qiu Y.-H. Peng Y.-P. (2020). Tgf-β1 provides neuroprotection via inhibition of microglial activation in 3-Acetylpyridine-induced cerebellar Ataxia model rats. Front. Neurosci. 14:187. doi: 10.3389/fnins.2020.00187 32265625 PMC7099147

[ref28] Carrasco E. Blum M. Weickert C. S. Casper D. (2003). Epidermal growth factor receptor expression is related to post-mitotic events in cerebellar development: regulation by thyroid hormone. Dev. Brain Res. 140, 1–13. doi: 10.1016/S0165-3806(02)00539-4, PMID: 12524172

[ref29] Carta I. Chen C. H. Schott A. L. Dorizan S. Khodakhah K. (2019). Cerebellar modulation of the reward circuitry and social behavior. Science 363:eaav0581. doi: 10.1126/science.aav0581 30655412 PMC6711161

[ref30] Carter A. R. Chen C. Schwartz P. M. Segal R. A. (2002). Brain-derived neurotrophic factor modulates cerebellar plasticity and synaptic ultrastructure. J. Neurosci. 22:1316. doi: 10.1523/JNEUROSCI.22-04-01316.2002, PMID: 11850459 PMC6757568

[ref31] Chang H. Riese Ii D. J. Gilbert W. Stern D. F. Mcmahan U. J. (1997). Ligands for ErbB-family receptors encoded by a neuregulin-like gene. Nature 387, 509–512. doi: 10.1038/387509a0, PMID: 9168114

[ref32] Chao M. V. Hempstead B. L. (1995). p75 and Trk: a two-receptor system. Trends Neurosci. 18, 321–326. doi: 10.1016/0166-2236(95)93922-K, PMID: 7571013

[ref33] Cheifetz S. Andres J. L. Massagué J. (1988). The transforming growth factor-beta receptor type iii is a membrane proteoglycan. Domain structure of the receptor. J. Biol. Chem. 263, 16984–16991. doi: 10.1016/S0021-9258(18)37487-8, PMID: 2903157

[ref34] Chen A. I. Zang K. Masliah E. Reichardt L. F. (2016). Glutamatergic axon-derived Bdnf controls Gabaergic synaptic differentiation in the cerebellum. Sci. Rep. 6:20201. doi: 10.1038/srep20201 26830657 PMC4735332

[ref35] Choo M. Miyazaki T. Yamazaki M. Kawamura M. Nakazawa T. Zhang J.. (2017). Retrograde Bdnf to TrkB signaling promotes synapse elimination in the developing cerebellum. Nat. Commun. 8:195. doi: 10.1038/s41467-017-00260-w 28775326 PMC5543168

[ref36] Clemow D. B. Steers W. D. Tuttle J. B. (2000). Stretch-activated signaling of nerve growth factor secretion in bladder and vascular smooth muscle cells from hypertensive and hyperactive rats. J. Cell. Physiol. 183, 289–300. doi: 10.1002/(SICI)1097-4652(200006)183:3<289::AID-JCP1>3.0.CO;2-6, PMID: 10797303

[ref37] Cohen-Cory S. Dreyfus C. F. Black I. B. (1991). Ngf and excitatory neurotransmitters regulate survival and morphogenesis of cultured cerebellar Purkinje cells. J. Neurosci. 11:462. doi: 10.1523/JNEUROSCI.11-02-00462.1991, PMID: 1671407 PMC6575229

[ref38] Cohen-Cory S. Elliott R. C. Dreyfus C. F. Black I. B. (1993). Depolarizing influences increase low-affinity Ngf receptor gene expression in cultured Purkinje neurons. Exp. Neurol. 119, 165–175. doi: 10.1006/exnr.1993.1018, PMID: 8094340

[ref39] Consalez G. G. Goldowitz D. Casoni F. Hawkes R. (2021). Origins, development, and compartmentation of the granule cells of the cerebellum. Front. Neural Circuits 14:611841. doi: 10.3389/fncir.2020.611841, PMID: 33519389 PMC7843939

[ref40] Constam D. B. Schmid P. Aguzzi A. Schachner M. Fontana A. (1994). Transient production of Tgf-β2 by postnatal cerebellar neurons and its effect on neuroblast proliferation. Eur. J. Neurosci. 6, 766–778. doi: 10.1111/j.1460-9568.1994.tb00988.x, PMID: 8075820

[ref41] Cook A. A. Jayabal S. Sheng J. Fields E. Leung T. C. S. Quilez S.. (2022). Activation of TrkB-Akt signaling rescues deficits in a mouse model of Sca6. Sci. Adv. 8:eabh3260. doi: 10.1126/sciadv.abh3260 36112675 PMC9481119

[ref42] Crowder R. J. Freeman R. S. (1998). Phosphatidylinositol 3-kinase and Akt protein kinase are necessary and sufficient for the survival of nerve growth factor-dependent sympathetic neurons. J. Neurosci. 18:2933. doi: 10.1523/JNEUROSCI.18-08-02933.1998, PMID: 9526010 PMC6792598

[ref43] Damarjian T. G. Craner M. J. Black J. A. Waxman S. G. (2004). Upregulation and colocalization of p75 and Nav1.8 in Purkinje neurons in experimental autoimmune encephalomyelitis. Neurosci. Lett. 369, 186–190. doi: 10.1016/j.neulet.2004.07.023, PMID: 15464262

[ref44] de Luca A. Weller M. Fontana A. (1996a). Tgf-beta-induced apoptosis of cerebellar granule neurons is prevented by depolarization. J. Neurosci. 16, 4174–4185. 8753879 10.1523/JNEUROSCI.16-13-04174.1996 PMC6578986

[ref45] de Luca A. Weller M. Frei K. Fontana A. (1996b). Maturation-dependent modulation of apoptosis in cultured cerebellar granule neurons by cytokines and Neurotrophins. Eur. J. Neurosci. 8, 1994–2005. 8921290 10.1111/j.1460-9568.1996.tb01343.x

[ref46] Di Marcotullio L. Ferretti E. De Smaele E. Screpanti I. Gulino A. (2006). Suppressors of hedgehog signaling. Mol. Neurobiol. 34, 193–204. doi: 10.1385/MN:34:3:193, PMID: 17308352

[ref47] Ding B. Dobner P. R. Mullikin-Kilpatrick D. Wang W. Zhu H. Chow C.-W.. (2018). Bdnf activates an Nfi-dependent neurodevelopmental timing program by sequestering Nfatc4. Mol. Biol. Cell 29, 975–987. doi: 10.1091/mbc.E16-08-0595, PMID: 29467254 PMC5896935

[ref48] Dobolyi A. Palkovits M. (2008). Expression of latent transforming growth factor beta binding proteins in the rat brain. J. Comp. Neurol. 507, 1393–1408. doi: 10.1002/cne.21621 18196529

[ref49] Doré J. J. E. Edens M. Garamszegi N. Leof E. B. (1998). Heteromeric and Homomeric transforming growth factor-β receptors show distinct signaling and endocytic responses in epithelial cells*. J. Biol. Chem. 273, 31770–31777. doi: 10.1074/jbc.273.48.31770, PMID: 9822641

[ref50] Doughty M. L. Lohof A. Campana A. Delhaye-Bouchaud N. Mariani J. (1998). Neurotrophin-3 promotes cerebellar granule cell exit from the Egl. Eur. J. Neurosci. 10, 3007–3011. doi: 10.1111/j.1460-9568.1998.00333.x, PMID: 9758170

[ref51] Elvers M. Pfeiffer J. Kaltschmidt C. Kaltschmidt B. (2005). Tgf-β2 neutralization inhibits proliferation and activates apoptosis of cerebellar granule cell precurors in the developing cerebellum. Mech. Dev. 122, 587–602. doi: 10.1016/j.mod.2004.10.012, PMID: 15804570

[ref52] Fantone S. Tossetta G. Montironi R. Senzacqua M. Marzioni D. Mazzucchelli R. (2020). Ciliary neurotrophic factor (Cntf) and its receptor (Cntfrα) signal through Mapk/Erk pathway in human prostate tissues: a morphological and biomolecular study. Europ. J. Histochem. 64:3147. doi: 10.4081/ejh.2020.3147 PMC7586252 33131268

[ref53] Fenster C. Vullhorst D. Buonanno A. (2012). Acute neuregulin-1 signaling influences Ampa receptor mediated responses in cultured cerebellar granule neurons. Brain Res. Bull. 87, 21–29. doi: 10.1016/j.brainresbull.2011.10.011, PMID: 22044943 PMC3432401

[ref54] Ferretti C. J. Hollander E. (2015). “The role of inflammation in autism Spectrum disorder” in Immunology and psychiatry: from basic research to therapeutic interventions. eds. Müller N. Myint A.-M. Schwarz M. J. (Cham: Springer International Publishing)

[ref55] Florez-Mcclure M. L. Linseman D. A. Chu C. T. Barker P. A. Bouchard R. J. Le S. S.. (2004). The p75 Neurotrophin receptor can induce autophagy and death of cerebellar Purkinje neurons. J. Neurosci. 24:4498. doi: 10.1523/JNEUROSCI.5744-03.2004, PMID: 15140920 PMC1876689

[ref56] Friedman W. J. (2000). Neurotrophins induce death of hippocampal neurons via the p75 receptor. J. Neurosci. 20:6340. doi: 10.1523/JNEUROSCI.20-17-06340.2000, PMID: 10964939 PMC6772976

[ref57] Frontera J. L. Baba Aissa H. Sala R. W. Mailhes-Hamon C. Georgescu I. A. Léna C.. (2020). Bidirectional control of fear memories by cerebellar neurons projecting to the ventrolateral periaqueductal grey. Nat. Commun. 11:5207. doi: 10.1038/s41467-020-18953-0 33060630 PMC7566591

[ref58] Gajendran N. Kapfhammer J. P. Lain E. Canepari M. Vogt K. Wisden W.. (2009). Neuregulin signaling is dispensable for Nmda- and Gaba(a)-receptor expression in the cerebellum in vivo. J. Neurosci. 29, 2404–2413. doi: 10.1523/JNEUROSCI.4303-08.2009, PMID: 19244516 PMC6666233

[ref59] Gao J.-H. Parsons L. M. Bower J. M. Xiong J. Li J. Fox P. T. (1996). Cerebellum implicated in sensory acquisition and discrimination rather than Motor control. Science 272, 545–547. doi: 10.1126/science.272.5261.545, PMID: 8614803

[ref60] Gao W. Q. Zheng J. L. Karihaloo M. (1995). Neurotrophin-4/5 (Nt-4/5) and brain-derived neurotrophic factor (Bdnf) act at later stages of cerebellar granule cell differentiation. J. Neurosci. 15:2656. doi: 10.1523/JNEUROSCI.15-04-02656.1995, PMID: 7722620 PMC6577773

[ref61] Ghosh A. Carnahan J. Greenberg M. E. (1994). Requirement for Bdnf in activity-dependent survival of cortical neurons. Science 263, 1618–1623. doi: 10.1126/science.7907431, PMID: 7907431

[ref62] Gilbertson R. J. Clifford S. C. Macmeekin W. Wright C. Perry R. H. Kelly P.. (1998). Expression of the ErbB-Neuregulin signaling network during human cerebellar development: implications for the biology of Medulloblastoma1. Cancer Res. 58, 3932–3941. PMID: 9731505

[ref63] Glass D. J. Nye S. H. Hantzopoulos P. Macchi M. J. Squinto S. P. Goldfarb M.. (1991). Trkl3 mediates Bdnf/Nt-3-dependent survival and proliferation in fibroblasts lacking the low affinity Ngf receptor. Cells 66, 405–413. doi: 10.1016/0092-8674(91)90629-D, PMID: 1649703

[ref64] Gómez-Pinilla F. Knauer D. J. Nieto-Sampedro M. (1988). Epidermal growth factor receptor immunoreactivity in rat brain development and cellular localization. Brain Res. 438, 385–390. doi: 10.1016/0006-8993(88)91369-8, PMID: 3345447

[ref65] Gorski J. A. Zeiler S. R. Tamowski S. Jones K. R. (2003). Brain-derived neurotrophic factor is required for the maintenance of cortical dendrites. J. Neurosci. 23, 6856–6865. doi: 10.1523/JNEUROSCI.23-17-06856.2003, PMID: 12890780 PMC6740724

[ref66] Guney E. Ceylan M. F. Kara M. Tekin N. Goker Z. Senses Dinc G.. (2014). Serum nerve growth factor (Ngf) levels in children with attention deficit/hyperactivity disorder (Adhd). Neurosci. Lett. 560, 107–111. doi: 10.1016/j.neulet.2013.12.026, PMID: 24361544

[ref67] Gunn-Moore F. J. Tavaré J. M. (1998). Apoptosis of cerebellar granule cells induced by serum withdrawal, glutamate or β-amyloid, is independent of Jun kinase or p38 mitogen activated protein kinase activation. Neurosci. Lett. 250, 53–56. doi: 10.1016/S0304-3940(98)00438-8, PMID: 9696064

[ref68] Heintz T. G. Eva R. Fawcett J. W. (2016). Regional regulation of Purkinje cell dendritic spines by Integrins and Eph/Ephrins. PLoS One 11:e0158558. doi: 10.1371/journal.pone.0158558, PMID: 27518800 PMC4982633

[ref69] Hempstead B. L. Salzer J. L. (2002). A Glial Spin on Neurotrophins. Science 298, 1184–1186. doi: 10.1126/science.1078709, PMID: 12424359

[ref70] Hu F. Padukkavidana T. Vægter C. B. Brady O. A. Zheng Y. Mackenzie I. R.. (2010). Sortilin-mediated endocytosis determines levels of the frontotemporal dementia protein, progranulin. Neuron 68, 654–667. doi: 10.1016/j.neuron.2010.09.034, PMID: 21092856 PMC2990962

[ref71] Huang E. J. Reichardt L. F. (2001). Neurotrophins: roles in neuronal development and function. Annu. Rev. Neurosci. 24, 677–736. doi: 10.1146/annurev.neuro.24.1.677, PMID: 11520916 PMC2758233

[ref72] Ichise T. Kano M. Hashimoto K. Yanagihara D. Nakao K. Shigemoto R.. (2000). mGluR1 in cerebellar Purkinje cells essential for long-term depression, synapse elimination, and motor coordination. Science 288, 1832–1835. doi: 10.1126/science.288.5472.1832, PMID: 10846166

[ref73] Ito M. (2006). Cerebellar circuitry as a neuronal machine. Prog. Neurobiol. 78, 272–303. doi: 10.1016/j.pneurobio.2006.02.006, PMID: 16759785

[ref74] Ito M. (2008). Control of mental activities by internal models in the cerebellum. Nat. Rev. Neurosci. 9, 304–313. doi: 10.1038/nrn2332, PMID: 18319727

[ref75] Jiang Y.-Y. Cao B.-B. Wang X.-Q. Peng Y.-P. Qiu Y.-H. (2015). Cerebellar ataxia induced by 3-Ap affects immunological function. Neuro Endocrinol. Lett. 36, 246–256. PMID: 26313392

[ref76] Joo W. Hippenmeyer S. Luo L. (2014). Dendrite morphogenesis depends on relative levels of Nt-3/TrkC signaling. Science 346, 626–629. doi: 10.1126/science.1258996, PMID: 25359972 PMC4631524

[ref77] Kane C. J. M. Brown G. J. Phelan K. D. (1996). Transforming growth factor-β2 both stimulates and inhibits neurogenesis of rat cerebellar granule cells in culture. Dev. Brain Res. 96, 46–51. doi: 10.1016/0165-3806(96)00092-2, PMID: 8922667

[ref78] Kano M. Hashimoto K. Kurihara H. Watanabe M. Inoue Y. Aiba A.. (1997). Persistent multiple climbing Fiber Innervationof cerebellar Purkinje Cellsin mice lacking mGluR1. Neuron 18, 71–79. doi: 10.1016/S0896-6273(01)80047-7, PMID: 9010206

[ref79] Kao A. W. Mckay A. Singh P. P. Brunet A. Huang E. J. (2017). Progranulin, lysosomal regulation and neurodegenerative disease. Nat. Rev. Neurosci. 18, 325–333. doi: 10.1038/nrn.2017.36, PMID: 28435163 PMC6040832

[ref80] Kaplan D. R. Miller F. D. (2000). Neurotrophin signal transduction in the nervous system. Curr. Opin. Neurobiol. 10, 381–391. doi: 10.1016/s0959-4388(00)00092-1 10851172

[ref81] Karam S. D. Burrows R. C. Logan C. Koblar S. Pasquale E. B. Bothwell M. (2000). Eph receptors and ephrins in the developing chick cerebellum: relationship to sagittal patterning and granule cell migration. J. Neurosci. 20, 6488–6500. doi: 10.1523/JNEUROSCI.20-17-06488.2000, PMID: 10964955 PMC6772988

[ref82] Katoh-Semba R. Takeuchi I. K. Semba R. Kato K. (2000). Neurotrophin-3 controls proliferation of granular precursors as well as survival of mature granule neurons in the developing rat Cerbellum. J. Neurochem. 74, 1923–1930. doi: 10.1046/j.1471-4159.2000.0741923.x, PMID: 10800935

[ref83] Katsu-Jiménez Y. Loría F. Corona J. C. Díaz-Nido J. (2016). Gene transfer of brain-derived neurotrophic factor (Bdnf) prevents neurodegeneration triggered by Fxn deficiency. Mol. Ther. 24, 877–889. doi: 10.1038/mt.2016.32, PMID: 26849417 PMC4881769

[ref84] Khursigara G. Bertin J. Yano H. Moffett H. Distefano P. S. Chao M. V. (2001). A Prosurvival function for the p75 receptor death domain mediated via the caspase recruitment domain receptor-interacting protein 2. J. Neurosci. 21:5854. doi: 10.1523/JNEUROSCI.21-16-05854.2001, PMID: 11487608 PMC6763175

[ref85] Kircher T. Thienel R. Wagner M. Reske M. Habel U. Kellermann T.. (2009). Neuregulin 1 ice-single nucleotide polymorphism in first episode schizophrenia correlates with cerebral activation in fronto-temporal areas. Eur. Arch. Psychiatry Clin. Neurosci. 259, 72–79. doi: 10.1007/s00406-008-0837-4, PMID: 18806920

[ref86] Kisiswa L. Fernández-Suárez D. Sergaki M. C. Ibáñez C. F. (2018). Rip2 gates Traf6 interaction with death receptor p75ntr to regulate cerebellar granule neuron survival. Cell Rep. 24, 1013–1024. doi: 10.1016/j.celrep.2018.06.098, PMID: 30044969

[ref87] Klesse L. J. Parada L. F. (1999). Trks: signal transduction and intracellular pathways. Microsc. Res. Tech. 45, 210–216. doi: 10.1002/(SICI)1097-0029(19990515/01)45:4/5<210::AID-JEMT4>3.0.CO;2-F, PMID: 10383113

[ref88] Kokubo M. Nishio M. Ribar T. J. Anderson K. A. West A. E. Means A. R. (2009). Bdnf-mediated cerebellar granule cell development is impaired in mice null for Camkk2 or Camkiv. J. Neurosci. 29:8901. doi: 10.1523/JNEUROSCI.0040-09.2009, PMID: 19605628 PMC2763571

[ref89] Koshimizu H. Hazama S. Hara T. Ogura A. Kojima M. (2010). Distinct signaling pathways of precursor Bdnf and mature Bdnf in cultured cerebellar granule neurons. Neurosci. Lett. 473, 229–232. doi: 10.1016/j.neulet.2010.02.055, PMID: 20219632

[ref90] Kostadinov D. Beau M. Blanco-Pozo M. Häusser M. (2019). Predictive and reactive reward signals conveyed by climbing fiber inputs to cerebellar Purkinje cells. Nat. Neurosci. 22, 950–962. doi: 10.1038/s41593-019-0381-8, PMID: 31036947 PMC7612392

[ref91] Kowanetz M. Valcourt U. Bergström R. Heldin C.-H. Moustakas A. (2004). Id2 and Id3 define the potency of cell proliferation and differentiation responses to transforming growth factor β and bone morphogenetic protein. Mol. Cell. Biol. 24, 4241–4254. doi: 10.1128/MCB.24.10.4241-4254.2004, PMID: 15121845 PMC400464

[ref92] Koziol L. F. Budding D. Andreasen N. D’arrigo S. Bulgheroni S. Imamizu H.. (2014). Consensus paper: the Cerebellum's role in movement and cognition. Cerebellum 13, 151–177. doi: 10.1007/s12311-013-0511-x, PMID: 23996631 PMC4089997

[ref93] Krainock R. Murphy S. (2001a). Heregulin upregulates the expression of nitric oxide synthase (Nos)-1 in rat cerebellar granule neurons via the ErbB4 receptor. J. Neurochem. 76, 312–315. doi: 10.1046/j.1471-4159.2001.00089.x 11146005

[ref94] Krainock R. Murphy S. (2001b). Regulation of functional nitric oxide synthase-1 expression in cerebellar granule neurons by heregulin is post-transcriptional, and involves mitogen-activated protein kinase. J. Neurochem. 78, 552–559. doi: 10.1046/j.1471-4159.2001.00420.x 11483658

[ref95] Kubo T. Nonomura T. Enokido Y. Hatanaka H. (1995). Brain-derived neurotrophic factor (bdnf) can prevent apoptosis of rat cerebellar granule neurons in culture. Dev. Brain Res. 85, 249–258. doi: 10.1016/0165-3806(94)00220-T, PMID: 7541321

[ref96] Lackey E. P. Sillitoe R. V. (2020). Eph/ephrin function contributes to the patterning of spinocerebellar mossy fibers into parasagittal zones. Front. Syst. Neurosci. 14:7. doi: 10.3389/fnsys.2020.00007, PMID: 32116578 PMC7033604

[ref97] Lärkfors L. Lindsay R. M. Alderson R. F. (1994). Ciliary neurotrophic factor enhances the survival of Purkinje cells in vitro. Eur. J. Neurosci. 6, 1015–1025. doi: 10.1111/j.1460-9568.1994.tb00596.x, PMID: 7952272

[ref98] Lärkfors L. Lindsay R. M. Alderson R. F. (1996). Characterization of the responses of Purkinje cells to Neurotrophin treatment. J. Neurochem. 66, 1362–1373. doi: 10.1046/j.1471-4159.1996.66041362.x, PMID: 8627287

[ref99] Larramendi L. M. H. Victor T. (1967). Synapses on the purkinje cell spines in the mouse an electronmicroscopic study. Brain Res. 5, 15–30. doi: 10.1016/0006-8993(67)90216-8, PMID: 6035937

[ref100] Lee R. Kermani P. Teng K. K. Hempstead B. L. (2001). Regulation of cell survival by secreted Proneurotrophins. Science 294, 1945–1948. doi: 10.1126/science.1065057, PMID: 11729324

[ref101] Leeds P. Leng Y. Chalecka-Franaszek E. Chuang D. M. (2005). Neurotrophins protect against cytosine arabinoside-induced apoptosis of immature rat cerebellar neurons. Neurochem. Int. 46, 61–72. doi: 10.1016/j.neuint.2004.07.001, PMID: 15567516

[ref102] Legrand C. Clos J. (1991). Biochemical, Immunocytochemical and morphological evidence for an interaction between thyroid hormone and nerve growth factor in the developing cerebellum of Normal and hypothyroid rats. Dev. Neurosci. 13, 382–396. doi: 10.1159/000112189, PMID: 1667097

[ref103] Leto K. Arancillo M. Becker E. B. E. Buffo A. Chiang C. Ding B.. (2016). Consensus Paper: Cerebellar Development. Cerebellum 15, 789–828. doi: 10.1007/s12311-015-0724-2, PMID: 26439486 PMC4846577

[ref104] Leutz A. Schachner M. (1981). Epidermal growth factor stimulates Dna-synthesis of astrocytes in primary cerebellar cultures. Cell Tissue Res. 220, 393–404. doi: 10.1007/BF00210517, PMID: 6271403

[ref105] Lin D.-S. Hsiao C.-D. Lee A. Y.-L. Ho C.-S. Liu H.-L. Wang T.-J.. (2015). Mitigation of cerebellar neuropathy in globoid cell leukodystrophy mice by Aav-mediated gene therapy. Gene 571, 81–90. doi: 10.1016/j.gene.2015.06.049, PMID: 26115766

[ref106] Lindholm D. Dechant G. Heisenberg C.-P. Thoenen H. (1993). Brain-derived neurotrophic factor is a survival factor for cultured rat cerebellar granule neurons and protects them against glutamate-induced neurotoxicity. Eur. J. Neurosci. 5, 1455–1464. doi: 10.1111/j.1460-9568.1993.tb00213.x, PMID: 7904521

[ref107] Lindholm D. Hamnér S. Zirrgiebel U. (1997). Neurotrophins and cerebellar development. Perspect. Dev. Neurobiol. 5, 83–94. PMID: 9509520

[ref108] Lu B. Pang P. T. Woo N. H. (2005). The yin and yang of neurotrophin action. Nat. Rev. Neurosci. 6, 603–614. doi: 10.1038/nrn1726, PMID: 16062169

[ref109] Mackie S. Shaw P. Lenroot R. Pierson R. Greenstein D. K. Nugent T. F.. (2007). Cerebellar development and clinical outcome in attention deficit hyperactivity disorder. Am. J. Psychiatr. 164, 647–655. doi: 10.1176/ajp.2007.164.4.647, PMID: 17403979

[ref110] Marino S. (2005). Medulloblastoma: developmental mechanisms out of control. Trends Mol. Med. 11, 17–22. doi: 10.1016/j.molmed.2004.11.008, PMID: 15649818

[ref111] Martinez R. Eller C. Viana N. B. Gomes F. C. A. (2011). Thyroid hormone induces cerebellar neuronal migration and Bergmann glia differentiation through epidermal growth factor/mitogen-activated protein kinase pathway. Eur. J. Neurosci. 33, 26–35. doi: 10.1111/j.1460-9568.2010.07490.x 21070391

[ref112] Matsui K. Furukawa S. Shibasaki H. Kikuchi T. (1990). Reduction of nerve growth factor level in the brain of genetically ataxic mice (weaver, reeler). FEBS Lett. 276, 78–80. doi: 10.1016/0014-5793(90)80511-G, PMID: 2265716

[ref113] Matsuwaki T. Kobayashi A. Mase K. Nakamura K. Nakano S.-I. Miyoshi T.. (2015). Possible involvement of the cerebellum in motor-function impairment in progranulin-deficient mice. Neuroreport 26:442. doi: 10.1097/WNR.0000000000000442 26302163

[ref114] Mcalhany R. E. Jr. Miranda R. C. Finnell R. H. West J. R. (1999). Ethanol decreases glial derived neurotrophic factor (Gdnf) protein release but not mrna expression and increases Gdnf-stimulated Shc phosphorylation in the developing cerebellum. Alcohol Clin. Exp. Res. 23, 1691–1697. doi: 10.1111/j.1530-0277.1999.tb04062.x, PMID: 10550003

[ref115] Mcalhany R. E. Jr. West J. R. Miranda R. C. (1997). Glial-derived neurotrophic factor rescues calbindin-D28k-immunoreactive neurons in alcohol-treated cerebellar explant cultures. J. Neurobiol. 33, 835–847. doi: 10.1002/(SICI)1097-4695(19971120)33:6<835::AID-NEU10>3.0.CO;2-3, PMID: 9369155

[ref116] Mellesmoen A. Sheeler C. Ferro A. Rainwater O. Cvetanovic M. (2019). Brain derived neurotrophic factor (Bdnf) delays onset of pathogenesis in transgenic mouse model of spinocerebellar Ataxia type 1 (Sca1). Front. Cell. Neurosci. 12:509. doi: 10.3389/fncel.2018.00509, PMID: 30718999 PMC6348256

[ref02] Melka M. G. Laufer B. I. Mcdonald P. Castellani C. A. Rajakumar N. O’reilly R.. (2014). The effects of olanzapine on genome-wide DNA methylation in the hippocampus and cerebellum. Clinical Epigenetics 6:1. 24382160 10.1186/1868-7083-6-1 PMC3895844

[ref117] Menshanov P. Lanshakov D. Dygalo N. (2015). Probdnf is a major product of bdnf gene expressed in the perinatal rat cortex. Physiol. Res. 64:996. doi: 10.33549/physiolres.932996 26047381

[ref118] Minichiello L. (1996). TrkB and TrkC neurotrophin receptors cooperate in promoting survival of hippocampal and cerebellar granule neurons. Genes Dev. 10, 2849–2858. doi: 10.1101/gad.10.22.2849, PMID: 8918886

[ref119] Moreno-Flores M. T. MartíN-Aparicio E. Ávila J. Díaz-Nido J. Wandosell F. (2002). Ephrin-B1 promotes dendrite outgrowth on cerebellar granule neurons. Mol. Cell. Neurosci. 20, 429–446. doi: 10.1006/mcne.2002.1128, PMID: 12139920

[ref120] Morrison R. S. Keating R. F. Moskal J. R. (1988). Basic fibroblast growth factor and epidermal growth factor exert differential trophic effects on Cns neurons. J. Neurosci. Res. 21, 71–79. doi: 10.1002/jnr.490210111, PMID: 3265159

[ref121] Morrison M. E. Mason C. A. (1998). Granule neuron regulation of Purkinje cell development: striking a balance between Neurotrophin and glutamate signaling. J. Neurosci. 18:3563. doi: 10.1523/JNEUROSCI.18-10-03563.1998, PMID: 9570788 PMC6793141

[ref122] Mount H. T. Dean D. O. Alberch J. Dreyfus C. F. Black I. B. (1995). Glial cell line-derived neurotrophic factor promotes the survival and morphologic differentiation of Purkinje cells. Proc. Natl. Acad. Sci. 92, 9092–9096. doi: 10.1073/pnas.92.20.9092, PMID: 7568079 PMC40930

[ref123] Mount H. T. J. Elkabes S. Dreyfus C. F. Black I. B. (1998). Differential involvement of metabotropic and p75 Neurotrophin receptors in effects of nerve growth factor and Neurotrophin-3 on cultured Purkinje cell survival. J. Neurochem. 70, 1045–1053. doi: 10.1046/j.1471-4159.1998.70031045.x, PMID: 9489724

[ref124] Muller Y. Duperray C. Caruso F. Clos J. (1994). Autocrine regulation of proliferation of cerebellar granule neurons by nerve growth factor. J. Neurosci. Res. 38, 41–55. doi: 10.1002/jnr.490380107, PMID: 8057389

[ref125] Murphy S. P. Bielby-Clarke K. (2008). Neuregulin signaling in neurons depends on ErbB4 interaction with Psd-95. Brain Res. 1207, 32–35. doi: 10.1016/j.brainres.2008.02.063, PMID: 18374309

[ref126] Neve A. Santhana Kumar K. Tripolitsioti D. Grotzer M. A. Baumgartner M. (2017). Investigation of brain tissue infiltration by medulloblastoma cells in an ex vivo model. Sci. Rep. 7:5297. doi: 10.1038/s41598-018-28051-3 28706234 PMC5509741

[ref127] Neveu I. Arenas E. (1996). Neurotrophins promote the survival and development of neurons in the cerebellum of hypothyroid rats in vivo. J. Cell Biol. 133, 631–646. doi: 10.1083/jcb.133.3.631, PMID: 8636237 PMC2120825

[ref128] Nickl-Jockschat T. Stöcker T. Krug A. Markov V. Huang R. Schneider F.. (2014). A Neuregulin-1 schizophrenia susceptibility variant causes perihippocampal fiber tract anomalies in healthy young subjects. Brain Behavior 4, 215–226. doi: 10.1002/brb3.203, PMID: 24683514 PMC3967537

[ref129] Nonomura T. Kubo T. Oka T. Shimoke K. Yamada M. Enokido Y.. (1996). Signaling pathways and survival effects of Bdnf and Nt-3 on cultured cerebellar granule cells. Dev. Brain Res. 97, 42–50. doi: 10.1016/S0165-3806(96)00130-7, PMID: 8946053

[ref130] Numakawa T. Nakayama H. Suzuki S. Kubo T. Nara F. Numakawa Y.. (2003). Nerve growth factor-induced glutamate release is via p75 receptor, ceramide, and Ca2+ from ryanodine receptor in developing cerebellar neurons*. J. Biol. Chem. 278, 41259–41269. doi: 10.1074/jbc.M304409200, PMID: 12902347

[ref131] Nykjaer A. Lee R. Teng K. K. Jansen P. Madsen P. Nielsen M. S.. (2004). Sortilin is essential for prongf-induced neuronal cell death. Nature 427, 843–848. doi: 10.1038/nature02319, PMID: 14985763

[ref132] Oh H. Fujio Y. Kunisada K. Hirota H. Matsui H. Kishimoto T.. (1998). Activation of phosphatidylinositol 3-kinase through glycoprotein 130 induces protein kinase B and p70 S6 kinase phosphorylation in cardiac myocytes*. J. Biol. Chem. 273, 9703–9710. 9545305 10.1074/jbc.273.16.9703

[ref133] Ohta M. Ohi T. Nishimura M. Itoh N. Hayashi K. Ohta K. (1996). Distribution of and age-related changes in ciliary neurotrophic factor protein in rat tissues. IUBMB Life 40:1273. doi: 10.1080/15216549600201273, PMID: 8950025

[ref134] Okano-Uchida T. Naruse M. Ikezawa T. Shibasaki K. Ishizaki Y. (2013). Cerebellar neural stem cells differentiate into two distinct types of astrocytes in response to Cntf and Bmp2. Neurosci. Lett. 552, 15–20. doi: 10.1016/j.neulet.2013.07.021, PMID: 23896528

[ref135] Ondáčová K. Jurkovičová D. Lacinová Ľ. (2017). Altered sodium and potassium, but not calcium currents in cerebellar granule cells in an in vitro model of neuronal injury. Cell. Mol. Neurobiol. 37, 771–782. doi: 10.1007/s10571-016-0416-6, PMID: 27517720 PMC11482125

[ref136] Ortega F. Pérez-Sen R. Morente V. Delicado E. G. Miras-Portugal M. T. (2010). P2X7, Nmda and Bdnf receptors converge on Gsk3 phosphorylation and cooperate to promote survival in cerebellar granule neurons. Cell. Mol. Life Sci. 67, 1723–1733. doi: 10.1007/s00018-010-0278-x, PMID: 20146080 PMC2858808

[ref137] Oyesiku N. M. Wigston D. J. (1996). Ciliary neurotrophic factor stimulates neurite outgrowth from spinal cord neurons. J. Comp. Neurol. 364, 68–77. doi: 10.1002/(SICI)1096-9861(19960101)364:1<68::AID-CNE6>3.0.CO;2-Q, PMID: 8789276

[ref138] Ozaki M. (2001). Neuregulins and the shaping of synapses. Neuroscientist 7, 146–154. doi: 10.1177/107385840100700209, PMID: 11496925

[ref139] Ozaki M. Itoh K. Miyakawa Y. Kishida H. Hashikawa T. (2004). Protein processing and releases of neuregulin-1 are regulated in an activity-dependent manner. J. Neurochem. 91, 176–188. doi: 10.1111/j.1471-4159.2004.02719.x, PMID: 15379898

[ref140] Ozaki M. Kishigami S. Yano R. (1998). Expression of receptors for neuregulins, ErbB2, ErbB3 and ErbB4, in developing mouse cerebellum. Neurosci. Res. 30, 351–354. 9678639 10.1016/s0168-0102(98)00013-3

[ref141] Ozaki M. Sasner M. Yano R. Lu H. S. Buonanno A. (1997). Neuregulin-β induces expression of an Nmda-receptor subunit. Nature 390, 691–694. doi: 10.1038/37795, PMID: 9414162

[ref142] Ozaki M. Tohyama K. Kishida H. Buonanno A. Yano R. Hashikawa T. (2000). Roles of neuregulin in synaptogenesis between mossy fibers and cerebellar granule cells. J. Neurosci. Res. 59, 612–623. doi: 10.1002/(SICI)1097-4547(20000301)59:5<612::AID-JNR4>3.0.CO;2-V, PMID: 10686589

[ref143] Paratcha G. Ledda F. (2008). Gdnf and Gfrα: a versatile molecular complex for developing neurons. Trends Neurosci. 31, 384–391. doi: 10.1016/j.tins.2008.05.003, PMID: 18597864

[ref144] Paushter D. H. Du H. Feng T. Hu F. (2018). The lysosomal function of progranulin, a guardian against neurodegeneration. Acta Neuropathol. 136, 1–17. doi: 10.1007/s00401-018-1861-8, PMID: 29744576 PMC6117207

[ref145] Peter S. Ten Brinke M. M. Stedehouder J. Reinelt C. M. Wu B. Zhou H.. (2016). Dysfunctional cerebellar Purkinje cells contribute to autism-like behaviour in Shank2-deficient mice. Nat. Commun. 7:12627. doi: 10.1038/ncomms12627 27581745 PMC5025785

[ref146] Pioro E. P. Claudio Cuello A. (1988). Purkinje cells of adult rat cerebellum express nerve growth factor receptor immunoreactivity: light microscopic observations. Brain Res. 455, 182–186. doi: 10.1016/0006-8993(88)90131-X, PMID: 2843259

[ref147] Proenca C. C. Song M. Lee F. S. (2016). Differential effects of Bdnf and neurotrophin 4 (Nt4) on endocytic sorting of TrkB receptors. J. Neurochem. 138, 397–406. doi: 10.1111/jnc.13676, PMID: 27216821 PMC4961580

[ref148] Rabacchi S. A. Kruk B. Hamilton J. Carney C. Hoffman J. R. Meyer S. L.. (1999). Bdnf and Nt4/5 promote survival and neurite outgrowth of pontocerebellar mossy fiber neurons. J. Neurobiol. 40, 254–269. doi: 10.1002/(SICI)1097-4695(199908)40:2<254::AID-NEU11>3.0.CO;2-4, PMID: 10413455

[ref149] Rahman A. Weber J. Labin E. Lai C. Prieto A. L. (2019). Developmental expression of Neuregulin-3 in the rat central nervous system. J. Comp. Neurol. 527, 797–817. doi: 10.1002/cne.24559 30328115

[ref01] Rakotomamonjy J. Ghoumari A. M. (2019). Brain-Derived Neurotrophic Factor Is Required for the Neuroprotective Effect of Mifepristone on Immature Purkinje Cells in Cerebellar Slice Culture. Int. J. Mol. Sci. Online, 20. 10.3390/ijms20020285 PMC6359295 30642045

[ref150] Rauskolb S. Zagrebelsky M. Dreznjak A. Deogracias R. Matsumoto T. Wiese S.. (2010). Global deprivation of brain-derived neurotrophic factor in the Cns reveals an area-specific requirement for dendritic growth. J. Neurosci. 30, 1739–1749. doi: 10.1523/JNEUROSCI.5100-09.2010 20130183 PMC6633992

[ref151] Reichardt L. F. (2006). Neurotrophin-regulated signalling pathways. Philos. Trans. Royal Soc. B Biol. Sci. 361, 1545–1564. doi: 10.1098/rstb.2006.1894, PMID: 16939974 PMC1664664

[ref152] Rico B. Xu B. Reichardt L. F. (2002). TrkB receptor signaling is required for establishment of Gabaergic synapses in the cerebellum. Nat. Neurosci. 5, 225–233. doi: 10.1038/nn808, PMID: 11836532 PMC2758226

[ref153] Rieff H. I. Corfas G. (2006). ErbB receptor signalling regulates dendrite formation in mouse cerebellar granule cells in vivo. Eur. J. Neurosci. 23, 2225–2229. doi: 10.1111/j.1460-9568.2006.04727.x, PMID: 16630068

[ref154] Rieff H. I. Raetzman L. T. Sapp D. W. Yeh H. H. Siegel R. E. Corfas G. (1999). Neuregulin induces Gaba(a) receptor subunit expression and neurite outgrowth in cerebellar granule cells. J. Neurosci. 19, 10757–10766. doi: 10.1523/JNEUROSCI.19-24-10757.1999, PMID: 10594059 PMC6784934

[ref155] Rio C. Rieff H. I. Qi P. Corfas G. (1997). Neuregulin and erbB receptors play a critical role in neuronal migration. Neuron 19, 39–50. 9247262 10.1016/s0896-6273(00)80346-3

[ref156] Rodger J. Salvatore L. Migani P. (2012). Should I stay or should I go? Ephs and Ephrins in neuronal migration. Neurosignals 20, 190–201. doi: 10.1159/000333784, PMID: 22456188

[ref157] Rogers J. H. Ciossek T. Menzel P. Pasquale E. B. (1999). Eph receptors and ephrins demarcate cerebellar lobules before and during their formation. Mech. Dev. 87, 119–128. doi: 10.1016/S0925-4773(99)00154-9, PMID: 10495276

[ref158] Roussel M. F. Hatten M. E. (2011). Cerebellum development and medulloblastoma. Curr. Top. Dev. Biol. 94, 235–282. doi: 10.1016/B978-0-12-380916-2.00008-5 21295689 PMC3213765

[ref159] Sadakata T. Furuichi T. (2009). Developmentally regulated Ca2+-dependent activator protein for secretion 2 (Caps2) is involved in Bdnf secretion and is associated with autism susceptibility. Cerebellum 8, 312–322. doi: 10.1007/s12311-009-0097-5, PMID: 19238500

[ref160] Sadakata T. Kakegawa W. Mizoguchi A. Washida M. Katoh-Semba R. Shutoh F.. (2007). Impaired cerebellar development and function in mice lacking Caps2, a protein involved in Neurotrophin release. J. Neurosci. 27:2472. doi: 10.1523/JNEUROSCI.2279-06.2007, PMID: 17344385 PMC6672497

[ref161] Sadakata T. Kakegawa W. Shinoda Y. Hosono M. Katoh-Semba R. Sekine Y.. (2014). Axonal localization of Ca2+-dependent activator protein for secretion 2 is critical for subcellular locality of brain-derived neurotrophic factor and Neurotrophin-3 release affecting proper development of postnatal mouse cerebellum. PLoS One 9:e99524. doi: 10.1371/journal.pone.0099524, PMID: 24923991 PMC4055771

[ref162] Sajdel-Sulkowska E. M. Xu M. Koibuchi N. (2009). Increase in cerebellar Neurotrophin-3 and oxidative stress markers in autism. Cerebellum 8, 366–372. doi: 10.1007/s12311-009-0105-9, PMID: 19357934

[ref163] Sajdel-Sulkowska E. M. Xu M. Mcginnis W. Koibuchi N. (2011). Brain region-specific changes in oxidative stress and Neurotrophin levels in autism Spectrum disorders (Asd). Cerebellum 10, 43–48. doi: 10.1007/s12311-010-0223-4, PMID: 20967576

[ref164] Sakuma K. Watanabe K. Totsuka T. Sano M. Nakano H. Nakao R.. (2002). The reciprocal change of neurotrophin-4 and glial cell line-derived neurotrophic factor protein in the muscles, spinal cord and cerebellum of the dy mouse. Acta Neuropathol. 104, 482–492. doi: 10.1007/s00401-002-0576-y, PMID: 12410396

[ref165] Sanchez-Perez A. Llansola M. Cauli O. Felipo V. (2005). Modulation of Nmda receptors in the cerebellum. Ii. Signaling pathways and physiological modulators regulating Nmda receptor function. Cerebellum 4, 162–170. doi: 10.1080/14734220510008003, PMID: 16147948

[ref166] Santhana Kumar K. Neve A. Guerreiro Stucklin A. S. Kuzan-Fischer C. M. Rushing E. J. Taylor M. D.. (2018). Tgf-β determines the pro-migratory potential of bfgf signaling in Medulloblastoma. Cell Rep. 23, 3798–3812.e8. doi: 10.1016/j.celrep.2018.05.083, PMID: 29949765

[ref167] Sathyanesan A. Zhou J. Scafidi J. Heck D. H. Sillitoe R. V. Gallo V. (2019). Emerging connections between cerebellar development, behaviour and complex brain disorders. Nat. Rev. Neurosci. 20, 298–313. doi: 10.1038/s41583-019-0152-2, PMID: 30923348 PMC7236620

[ref168] Saywell V. Cioni J.-M. Ango F. (2014). Developmental gene expression profile of axon guidance cues in Purkinje cells during cerebellar circuit formation. Cerebellum 13, 307–317. doi: 10.1007/s12311-014-0548-5, PMID: 24550128

[ref169] Scalabrino G. (2022). Epidermal growth factor in the Cns: a beguiling journey from integrated cell biology to multiple sclerosis. An extensive translational overview. Cell. Mol. Neurobiol. 42, 891–916. doi: 10.1007/s10571-020-00989-x, PMID: 33151415 PMC8942922

[ref170] Schmahmann J. D. (2004). Disorders of the cerebellum: Ataxia, Dysmetria of thought, and the cerebellar cognitive affective syndrome. J. Neuropsychiatry Clin. Neurosci. 16, 367–378. doi: 10.1176/jnp.16.3.367, PMID: 15377747

[ref171] Schmahmann J. D. Caplan D. (2006). Cognition, emotion and the cerebellum. Brain 129, 290–292. doi: 10.1093/brain/awh729, PMID: 16434422

[ref172] Schmahmann J. D. Sherman J. C. (1998). The cerebellar cognitive affective syndrome. Brain 121, 561–579. doi: 10.1093/brain/121.4.561, PMID: 9577385

[ref173] Schmitt A. Koschel J. Zink M. Bauer M. Sommer C. Frank J.. (2010). Gene expression of Nmda receptor subunits in the cerebellum of elderly patients with schizophrenia. Eur. Arch. Psychiatry Clin. Neurosci. 260, 101–111. doi: 10.1007/s00406-009-0017-1, PMID: 19856012 PMC2830629

[ref174] Schönholzer M. T. Migliavacca J. Alvarez E. Santhana Kumar K. Neve A. Gries A.. (2020). Real-time sensing of Mapk signaling in medulloblastoma cells reveals cellular evasion mechanism counteracting dasatinib blockade of Erk activation during invasion. Neoplasia 22, 470–483. doi: 10.1016/j.neo.2020.07.006, PMID: 32818841 PMC7452206

[ref175] Schwartz P. M. Borghesani P. R. Levy R. L. Pomeroy S. L. Segal R. A. (1997). Abnormal cerebellar development and foliation in Bdnf−/− mice reveals a role for Neurotrophins in Cns patterning. Neuron 19, 269–281. doi: 10.1016/S0896-6273(00)80938-1, PMID: 9292718

[ref176] Segal R. A. Takahashi H. Mckay R. D. G. (1992). Changes in neurotrophin responsiveness during the development of cerebellar granule neurons. Neuron 9, 1041–1052. doi: 10.1016/0896-6273(92)90064-K, PMID: 1463606

[ref177] Seil F. J. (1999). Bdnf and Nt-4, but not Nt-3, promote development of inhibitory synapses in the absence of neuronal activity. Brain Res. 818, 561–564. doi: 10.1016/S0006-8993(98)01304-3, PMID: 10082848

[ref178] Seil F. J. Drake-Baumann R. (2000). TrkB receptor ligands promote activity-dependent inhibitory synaptogenesis. J. Neurosci. 20:5367. doi: 10.1523/JNEUROSCI.20-14-05367.2000, PMID: 10884321 PMC6772333

[ref179] Sentürk A. Pfennig S. Weiss A. Burk K. Acker-Palmer A. (2011). Ephrin Bs are essential components of the Reelin pathway to regulate neuronal migration. Nature 472, 356–360. doi: 10.1038/nature09874, PMID: 21460838

[ref180] Sergaki M. C. Ibáñez C. F. (2017). Gfrα1 regulates Purkinje cell migration by counteracting Ncam function. Cell Rep. 18, 367–379. doi: 10.1016/j.celrep.2016.12.039, PMID: 28076782 PMC5263233

[ref181] Seroogy K. B. Gall C. M. Lee D. C. Kornblum H. I. (1995). Proliferative zones of postnatal rat brain express epidermal growth factor receptor mrna. Brain Res. 670, 157–164. doi: 10.1016/0006-8993(94)01300-7, PMID: 7719717

[ref182] Shelton D. L. Reichardt L. F. (1986). Studies on the expression of the beta nerve growth factor (Ngf) gene in the central nervous system: level and regional distribution of Ngf mrna suggest that Ngf functions as a trophic factor for several distinct populations of neurons. Proc. Natl. Acad. Sci. 83, 2714–2718. doi: 10.1073/pnas.83.8.2714, PMID: 3458230 PMC323370

[ref183] Sherrard R. M. Bower A. J. (2002). Climbing fiber development: do neurotrophins have a part to play? Cerebellum 1, 265–275. doi: 10.1080/147342202320883579, PMID: 12879965

[ref184] Shim S.-H. Hwangbo Y. Yoon H.-J. Kwon Y.-J. Lee H.-Y. Hwang J.-A.. (2015). Increased levels of plasma glial-derived neurotrophic factor in children with attention deficit hyperactivity disorder. Nord. J. Psychiatry 69, 546–551. doi: 10.3109/08039488.2015.1014834, PMID: 25753832

[ref185] Shimada A. Mason C. A. Morrison M. E. (1998). TrkB signaling modulates spine density and morphology independent of dendrite structure in cultured neonatal Purkinje cells. J. Neurosci. 18:8559. doi: 10.1523/JNEUROSCI.18-21-08559.1998, PMID: 9786964 PMC6793520

[ref186] Shimoke K. Kubo T. Numakawa T. Abiru Y. Enokido Y. Takei N.. (1997). Involvement of phosphatidylinositol-3 kinase in prevention of low K+-induced apoptosis of cerebellar granule neurons. Dev. Brain Res. 101, 197–206. doi: 10.1016/S0165-3806(97)00065-5, PMID: 9263593

[ref187] Shinoda Y. Sadakata T. Yagishita K. Kinameri E. Katoh-Semba R. Sano Y.. (2019). Aspects of excitatory/inhibitory synapses in multiple brain regions are correlated with levels of brain-derived neurotrophic factor/neurotrophin-3. Biochem. Biophys. Res. Commun. 509, 429–434. doi: 10.1016/j.bbrc.2018.12.100, PMID: 30594389

[ref188] Simonati A. Williams R. E. (2022). Neuronal ceroid Lipofuscinosis: the multifaceted approach to the clinical issues, an overview. Front. Neurol. 13:811686. doi: 10.3389/fneur.2022.811686, PMID: 35359645 PMC8961688

[ref189] Singh K. K. Park K. J. Hong E. J. Kramer B. M. Greenberg M. E. Kaplan D. R.. (2008). Developmental axon pruning mediated by Bdnf-p75ntr–dependent axon degeneration. Nat. Neurosci. 11, 649–658. doi: 10.1038/nn.2114, PMID: 18382462

[ref190] Skaper S. D. Floreani M. Negro A. Facci L. Giusti P. (1998). Neurotrophins rescue cerebellar granule neurons from oxidative stress-mediated apoptotic death: selective involvement of phosphatidylinositol 3-kinase and the mitogen-activated protein kinase pathway. J. Neurochem. 70, 1859–1868. doi: 10.1046/j.1471-4159.1998.70051859.x, PMID: 9572269

[ref191] Snider R. S. Maiti A. Snider S. R. (1976). Cerebellar pathways to ventral midbrain and nigra. Exp. Neurol. 53, 714–728. doi: 10.1016/0014-4886(76)90150-3, PMID: 1001395

[ref192] Soontornniyomkij B. Everall I. P. Chana G. Tsuang M. T. Achim C. L. Soontornniyomkij V. (2011). Tyrosine kinase B protein expression is reduced in the cerebellum of patients with bipolar disorder. J. Affect. Disord. 133, 646–654. doi: 10.1016/j.jad.2011.04.044, PMID: 21612826 PMC3163025

[ref193] Stoodley C. J. (2012). The cerebellum and cognition: evidence from functional imaging studies. Cerebellum 11, 352–365. doi: 10.1007/s12311-011-0260-7, PMID: 21373864

[ref194] Stoodley C. J. (2016). The cerebellum and neurodevelopmental disorders. Cerebellum 15, 34–37. doi: 10.1007/s12311-015-0715-3, PMID: 26298473 PMC4811332

[ref195] Strick P. L. Dum R. P. Fiez J. A. (2009). Cerebellum and nonmotor function. Annu. Rev. Neurosci. 32, 413–434. doi: 10.1146/annurev.neuro.31.060407.125606, PMID: 19555291

[ref196] Subramaniam S. Strelau J. Unsicker K. (2008). Gdnf prevents Tgf-β-induced damage of the plasma membrane in cerebellar granule neurons by suppressing activation of p38-Mapk via the phosphatidylinositol 3-kinase pathway. Cell Tissue Res. 331, 373–383. doi: 10.1007/s00441-007-0538-8, PMID: 18071753

[ref197] Syed Z. Dudbridge F. Kent L. (2007). An investigation of the neurotrophic factor genes Gdnf, Ngf, and Nt3 in susceptibility to Adhd. Am. J. Med. Genet. B Neuropsychiatr. Genet. 144B, 375–378. doi: 10.1002/ajmg.b.30459, PMID: 17192954

[ref198] Takumi K. Mori T. Shimizu K. Hayashi M. (2005). Developmental changes in concentrations and distributions of neurotrophins in the monkey cerebellar cortex. J. Chem. Neuroanat. 30, 212–220. doi: 10.1016/j.jchemneu.2005.08.004, PMID: 16219447

[ref199] Tanaka S. Sekino Y. Shirao T. (2000). The effects of neurotrophin-3 and brain-derived neurotrophic factor on cerebellar granule cell movement and neurite extension in vitro. Neuroscience 97, 727–734. doi: 10.1016/S0306-4522(00)00049-X, PMID: 10842017

[ref200] Tang W. Lu Y. Tian Q.-Y. Zhang Y. Guo F.-J. Liu G.-Y.. (2011). The growth factor progranulin binds to Tnf receptors and is therapeutic against inflammatory arthritis in mice. Science 332, 478–484. doi: 10.1126/science.1199214 21393509 PMC3104397

[ref201] Tepper B. Bartkowska K. Okrasa M. Ngati S. Braszak M. Turlejski K.. (2020). Downregulation of TrkC receptors increases dendritic Arborization of Purkinje cells in the developing cerebellum of the opossum, *Monodelphis domestica*. Front. Neuroanat. 14:614617. doi: 10.3389/fnana.2020.614617, PMID: 33240051 PMC7677404

[ref202] Tiveron C. Fasulo L. Capsoni S. Malerba F. Marinelli S. Paoletti F.. (2013). Prongf\Ngf imbalance triggers learning and memory deficits, neurodegeneration and spontaneous epileptic-like discharges in transgenic mice. Cell Death Differ. 20, 1017–1030. doi: 10.1038/cdd.2013.22, PMID: 23538417 PMC3705592

[ref203] Tong L. Perez-Polo R. (1998). Brain-derived neurotrophic factor (Bdnf) protects cultured rat cerebellar granule neurons against glucose deprivation-induced apoptosis. J. Neural Transm. 105, 905–914. doi: 10.1007/s007020050101, PMID: 9869325

[ref204] Townley R. A. Boeve B. F. Benarroch E. E. (2018). Progranulin: functions and neurologic correlations. Neurology 90, 118–125. doi: 10.1212/WNL.0000000000004840, PMID: 29263224 PMC5772162

[ref205] Treanor J. J. S. Goodman L. De Sauvage F. Stone D. M. Poulsen K. T. Beck C. D.. (1996). Characterization of a multicomponent receptor for Gdnf. Nature 382, 80–83. doi: 10.1038/382080a0, PMID: 8657309

[ref206] Trupp M. Arenas E. Fainzilber M. Nilsson A.-S. Sieber B.-A. Grigoriou M.. (1996). Functional receptor for Gdnf encoded by the c-ret proto-oncogene. Nature 381, 785–789. doi: 10.1038/381785a0, PMID: 8657281

[ref207] Tunca Z. Kıvırcık Akdede B. Özerdem A. Alkın T. Polat S. Ceylan D.. (2015). Diverse glial cell line-derived neurotrophic factor (Gdnf) support between mania and schizophrenia: a comparative study in four major psychiatric disorders. Eur. Psychiatry 30, 198–204. doi: 10.1016/j.eurpsy.2014.11.003, PMID: 25543333

[ref208] Uesaka N. Abe M. Konno K. Yamazaki M. Sakoori K. Watanabe T.. (2018). Retrograde signaling from Progranulin to Sort1 counteracts synapse elimination in the developing cerebellum. Neuron 97, 796–805.e5. doi: 10.1016/j.neuron.2018.01.018, PMID: 29398357

[ref209] Van Damme P. Van Hoecke A. Lambrechts D. Vanacker P. Bogaert E. Van Swieten J.. (2008). Progranulin functions as a neurotrophic factor to regulate neurite outgrowth and enhance neuronal survival. J. Cell Biol. 181, 37–41. doi: 10.1083/jcb.200712039, PMID: 18378771 PMC2287280

[ref210] Vicario A. Kisiswa L. Tann J. Y. Kelly C. E. Ibáñez C. F. (2015). Neuron-type-specific signaling by the p75ntr death receptor is regulated by differential proteolytic cleavage. J. Cell Sci. 128, 1507–1517. doi: 10.1242/jcs.161745, PMID: 25720379

[ref211] Voisin A. Damon-Soubeyrand C. Bravard S. Saez F. Drevet J. R. Guiton R. (2020). Differential expression and localisation of Tgf-β isoforms and receptors in the murine epididymis. Sci. Rep. 10:995. doi: 10.1038/s41598-020-57839-5 31969637 PMC6976608

[ref212] Wagner M. J. Kim T. H. Savall J. Schnitzer M. J. Luo L. (2017). Cerebellar granule cells encode the expectation of reward. Nature 544, 96–100. doi: 10.1038/nature21726, PMID: 28321129 PMC5532014

[ref213] Wang L. Chen J. Hu Y. Liao A. Zheng W. Wang X.. (2022). Progranulin improves neural development via the Pi3K/Akt/Gsk-3β pathway in the cerebellum of a Vpa-induced rat model of Asd. Transl. Psychiatry 12:114. doi: 10.1038/s41398-022-01875-4 35318322 PMC8941112

[ref214] Wang W. Mullikin-Kilpatrick D. Crandall J. E. Gronostajski R. M. Litwack E. D. Kilpatrick D. L. (2007). Nuclear factor I coordinates multiple phases of cerebellar granule cell development via regulation of cell adhesion molecules. J. Neurosci. 27, 6115–6127. doi: 10.1523/JNEUROSCI.0180-07.2007, PMID: 17553984 PMC6672151

[ref215] Wang L. Nomura M. Goto Y. Tanaka K. Sakamoto R. Abe I.. (2011). Smad2 protein disruption in the central nervous system leads to aberrant cerebellar development and early postnatal Ataxia in mice*. J. Biol. Chem. 286, 18766–18774. doi: 10.1074/jbc.M111.223271, PMID: 21464123 PMC3099693

[ref216] Wang X. Yue T.-L. White R. F. Barone F. C. Feuerstein G. Z. (1995). Transforming growth factor-β1 exhibits delayed gene expression following focal cerebral ischemia. Brain Res. Bull. 36, 607–609. doi: 10.1016/0361-9230(94)00243-T, PMID: 7757496

[ref217] Wee P. Wang Z. (2017). Epidermal growth factor receptor cell proliferation signaling pathways. Cancers 9:52. doi: 10.3390/cancers9050052, PMID: 28513565 PMC5447962

[ref218] Wieduwilt M. J. Moasser M. M. (2008). The epidermal growth factor receptor family: biology driving targeted therapeutics. Cell. Mol. Life Sci. 65, 1566–1584. doi: 10.1007/s00018-008-7440-8, PMID: 18259690 PMC3060045

[ref219] Wong R. W. C. Guillaud L. (2004). The role of epidermal growth factor and its receptors in mammalian Cns. Cytokine Growth Factor Rev. 15, 147–156. doi: 10.1016/j.cytogfr.2004.01.004, PMID: 15110798

[ref220] Xie F. Padival M. Siegel R. E. (2007). Association of Psd-95 with ErbB4 facilitates neuregulin signaling in cerebellar granule neurons in culture. J. Neurochem. 100, 62–72. doi: 10.1111/j.1471-4159.2006.04182.x, PMID: 17074065

[ref221] Xie F. Raetzman L. T. Siegel R. E. (2004). Neuregulin induces Gabaa receptor β2 subunit expression in cultured rat cerebellar granule neurons by activating multiple signaling pathways. J. Neurochem. 90, 1521–1529. doi: 10.1111/j.1471-4159.2004.02685.x, PMID: 15341535

[ref222] Xu X. Wu D. Hou S. Zhu J. Li J. Tang J. (2017). Prenatal exposure to Tak242 affects the childhood autism in offspring in animal models of autism spectrum disorder. Iran. J. Basic Med. Sci. 20, 1016–1020. doi: 10.22038/IJBMS.2017.9270, PMID: 29085596 PMC5651454

[ref223] Yacubova E. Komuro H. (2002). Cellular and molecular mechanisms of cerebellar granule cell migration. Cell Biochem. Biophys. 37, 213–234. doi: 10.1385/cbb:37:3:213 12625628

[ref224] Yamada M. Ikeuchi T. Hatanaka H. (1997). The neurotrophic action and signalling of epidermal growth factor. Prog. Neurobiol. 51, 19–37. doi: 10.1016/S0301-0082(96)00046-9, PMID: 9044427

[ref225] Yamashita K. Gerken U. Vogel P. Hossmann K. A. Wiessner C. (1999). Biphasic expression of Tgf-β1 mrna in the rat brain following permanent occlusion of the middle cerebral artery. Brain Res. 836, 139–145. doi: 10.1016/S0006-8993(99)01626-1, PMID: 10415412

[ref226] Yamashita N. Mosinger B. Roy A. Miyazaki M. Ugajin K. Nakamura F.. (2011). Crmp5 (Collapsin response mediator protein 5) regulates dendritic development and synaptic plasticity in the cerebellar Purkinje cells. J. Neurosci. 31:1773. doi: 10.1523/JNEUROSCI.5337-10.2011, PMID: 21289187 PMC6623753

[ref227] Yang B. Ren Q. Zhang J. C. Chen Q. X. Hashimoto K. (2017). Altered expression of Bdnf, Bdnf pro-peptide and their precursor probdnf in brain and liver tissues from psychiatric disorders: rethinking the brain–liver axis. Transl. Psychiatry 7:e1128. doi: 10.1038/tp.2017.95, PMID: 28509900 PMC5534963

[ref228] Yeganeh-Doost P. Gruber O. Falkai P. Schmitt A. (2011). The role of the cerebellum in schizophrenia: from cognition to molecular pathways. Clinics 66, 71–77. doi: 10.1590/S1807-59322011001300009 21779725 PMC3118440

[ref229] Yoon S. O. Casaccia-Bonnefil P. Carter B. Chao M. V. (1998). Competitive signaling between TrkA and p75 nerve growth factor receptors determines cell survival. J. Neurosci. 18:3273. doi: 10.1523/JNEUROSCI.18-09-03273.1998, PMID: 9547236 PMC6792655

[ref230] Zanin J. P. Abercrombie E. Friedman W. J. (2016). Proneurotrophin-3 promotes cell cycle withdrawal of developing cerebellar granule cell progenitors via the p75 neurotrophin receptor. elife 5:e16654. doi: 10.7554/eLife.16654, PMID: 27434667 PMC4975574

[ref231] Zanin J. P. Friedman W. J. (2022). p75ntr prevents the onset of cerebellar granule cell migration via RhoA activation. elife 11:e79934. doi: 10.7554/eLife.79934, PMID: 36040414 PMC9427104

[ref232] Zanin J. P. Verpeut J. L. Li Y. Shiflett M. W. Wang S. S. H. Santhakumar V.. (2019). The p75ntr influences cerebellar circuit development and adult behavior via regulation of cell cycle duration of granule cell progenitors. J. Neurosci. 39:9119. doi: 10.1523/JNEUROSCI.0990-19.2019, PMID: 31582529 PMC6855675

[ref233] Zhang Y. Alexander P. Wang X.-F. (2016). Tgf-β family signaling in the control of cell proliferation and survival. Cold Spring Harb. Perspect. Biol. 9:a022145. doi: 10.1101/cshperspect.a022145 PMC5378054 27920038

[ref234] Zhang L. Goldman J. E. (1996). Generation of cerebellar interneurons from dividing progenitors in White matter. Neuron 16, 47–54. doi: 10.1016/S0896-6273(00)80022-7, PMID: 8562089

[ref235] Zhou P. Porcionatto M. Pilapil M. Chen Y. Choi Y. Tolias K. F.. (2007). Polarized signaling endosomes coordinate Bdnf-induced chemotaxis of cerebellar precursors. Neuron 55, 53–68. doi: 10.1016/j.neuron.2007.05.030, PMID: 17610817 PMC2661852

[ref236] Zhou X. Sun L. Bastos De Oliveira F. Qi X. Brown W. J. Smolka M. B.. (2015). Prosaposin facilitates sortilin-independent lysosomal trafficking of progranulin. J. Cell Biol. 210, 991–1002. doi: 10.1083/jcb.201502029, PMID: 26370502 PMC4576858

[ref237] Zhou Y.-X. Zhao M. Li D. Shimazu K. Sakata K. Deng C.-X.. (2003). Cerebellar deficits and hyperactivity in mice lacking Smad4*. J. Biol. Chem. 278, 42313–42320. doi: 10.1074/jbc.M308287200, PMID: 12896967

[ref238] Zirrgiebel U. Ohga Y. Carter B. Berninger B. Inagaki N. Thoenen H.. (1995). Characterization of TrkB receptor-mediated signaling pathways in rat cerebellar granule neurons: involvement of protein kinase C in neuronal survival. J. Neurochem. 65, 2241–2250. doi: 10.1046/j.1471-4159.1995.65052241.x, PMID: 7595513

